# Non-Coding RNA Profile in the Progression of Carotid Atherosclerosis: A Systematic Review

**DOI:** 10.3390/ijms27021002

**Published:** 2026-01-19

**Authors:** Gemma Sardelli, Pasquale Bufano, Rosetta Ragusa, Marco Laurino, Gabriele Masini, Luna Gargani, Danilo Neglia, Raffaele De Caterina, Chiara Caselli

**Affiliations:** 1 Institute of Clinical Physiology, Consiglio Nazionale delle Ricerche (CNR), 56124 Pisa, Italy; gemmasardelli@cnr.it (G.S.); pasqualebufano@cnr.it (P.B.); rosettaragusa@cnr.it (R.R.); marco.laurino@cnr.it (M.L.); 2 Department of Surgical, Medical and Molecular Pathology and Critical Care Medicine, University of Pisa, 56126 Pisa, Italy; 3 University of Pisa, Cardiovascular Division 1, Pisa University Hospital, 56124 Pisa, Italy; gabriele.masini@unipi.it (G.M.); luna.gargani@unipi.it (L.G.); raffaele.decaterina@unipi.it (R.D.C.); 4 Fondazione Toscana Gabriele Monasterio, 56124 Pisa, Italy; dneglia@ftgm.it

**Keywords:** carotid atherosclerosis, carotid plaque, ischemic stroke, non-coding RNA, biomarkers

## Abstract

Carotid atherosclerosis remains one of the primary etiological factors underlying ischemic stroke, contributing to adult neurological disability and mortality. In recent years, non-coding RNAs (ncRNAs) have emerged as key regulators of gene expression, actively modulating molecular pathways involved in atherogenesis. This systematic review, the first to be exclusively focused on carotid atherosclerosis, aimed at synthesizing current findings on the differential expression of ncRNAs throughout the natural history of the disease, thus providing the first comprehensive attempt to delineate a stage-specific ncRNA expression profile in carotid disease. A comprehensive literature search was conducted in PubMed and Scopus databases in January 2025, following PRISMA guidelines. Original studies involving human subjects with carotid atherosclerosis, evaluating the expression of intracellular or circulating ncRNAs, were included and then categorized according to their association with cardiovascular risk factors, carotid intima-media thickness (cIMT), presence of atherosclerotic plaques, plaque vulnerability, clinical symptoms, and ischemic stroke. Out of 148 articles initially identified, 49 met the inclusion criteria and were analyzed in depth. Among the different classes of ncRNAs, microRNAs (miRNAs) were the most frequently reported as dysregulated, followed by circular RNAs (circRNAs) and long non-coding RNAs (lncRNAs). Notably, the majority of identified ncRNAs were implicated in key pathogenic mechanisms such as inflammatory signaling, vascular smooth muscle cell (VSMC) phenotypic modulation, and ABCA1-mediated cholesterol efflux. Collectively, the evidence underscores the association and possible involvement of ncRNAs in the initiation and progression of carotid atherosclerosis and its cerebrovascular complications. Their relative stability in biological fluids and cell-specific expression profiles highlight their strong potential as minimally invasive biomarkers and—possibly—novel therapeutic targets.

## 1. Introduction

Atherosclerosis is a chronic, multifactorial disease of the arterial system, characterized by the progressive thickening of the innermost layer of artery—the intima media—accompanied by reduced elasticity and impaired blood flow [1]. Atherosclerotic lesions exhibit site-specific predilection, predominantly occurring at arterial branch points and curvatures subjected to disturbed flow dynamics and low endothelial shear stress, which promote endothelial dysfunction and plaque initiation [2]. Commonly, these atheroprone areas include carotid bifurcation, coronary arteries, abdominal aorta, and peripheral arteries such as iliac and femoral arteries [2]. Carotid atherosclerosis refers to the manifestation of this disease in cervical arteries and is associated with increased risk for stroke, transient ischemic attack (TIA) and vascular dementia [3].

**Figure 1:**
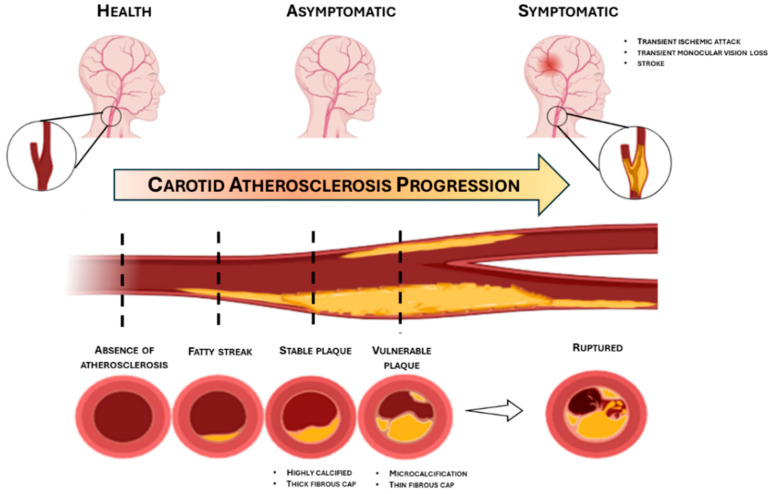
Natural history of carotid plaque progression. From left to right: normal carotid lumen representative of healthy subjects; early fatty streak evolving into a mature atheromatous plaque, typically observed in asymptomatic patients; vulnerable plaque prone to rupture leading to symptoms and stroke. Image created with BioRender.com.

Traditionally, the initiation of atherosclerosis has been ascribed to the accumulation of lipoproteins, recruitment of inflammatory cells [4], endothelial dysfunction [5], and vascular smooth muscle cells (VSMC) dysregulation [6]. At a cellular level, various predisposing factors, e.g., genetic background, metabolic disorders, hypertension, sedentary lifestyle and tobacco exposure, may reduce the ability of endothelial cells to produce nitric oxide (NO), crucial for controlling vascular tone, platelet adhesion, coagulation and immune function, thus promoting endothelial dysfunction [7,8]. In turn, the dysfunctional endothelium adopts a pro-inflammatory and pro-thrombotic phenotype, secreting cytokines, chemokines, and adhesion molecules that exacerbate local oxidative stress and promote leukocyte transmigration [9]. Endothelial activation leads to the upregulation of adhesion molecules, such as vascular cell adhesion molecules (VCAM), intracellular adhesion molecules (ICAM) and selectins, facilitating the adhesion of circulating monocytes and lymphocytes, as well as the endothelial permeability, allowing the entry of low-density lipoproteins (LDL)-cholesterol or monocytes through the endothelium [7]. Once in the subendothelial space, monocytes differentiate into macrophages that internalize oxidized LDL, giving rise to “foam cells” and further amplifying local inflammation. In parallel, VSMCs, which constitute the tunica media, undergo phenotyping switching, acquiring increased secretory, migratory and proliferative capabilities. Additionally, VSMCs can also internalize oxidized LDL and contribute to foam cell formation [10]. Inflammatory mediators released by VSMCs—such as interleukin (IL)-1β, IL-8, tumor necrosis factor (TNF)-α and β, IL-6, monocyte chemoattractant factor-1 (MCP-1) and CD-40 ligand—further promote immune cell recruitment and enhance extracellular matrix deposition, which is considered a key event in the initiation of carotid plaque [8]. Carotid plaque progresses from initial fatty streak to mature atheromatous plaque, mainly comprising a lipid-rich necrotic core (LRNC), extracellular matrix components, inflammatory infiltrates and foam cells, with a matrix-rich fibrous cap developing all around and protruding toward the arterial lumen [11] (Figure 1). Progressive cell death and defective efferocytosis contribute to core enlargement and destabilization. Plaques with large LRNCs, thin fibrous caps, intraplaque hemorrhage, or microcalcifications are considered vulnerable and prone to rupture. In the carotid territory, rupture or ulceration of such plaques can precipitate thromboembolism and acute cerebrovascular events.

The pathophysiological cascade of carotid atherosclerosis—from endothelial perturbation to plaque destabilization and ischemic strokes—is orchestrated by a complex network of molecular regulators. Among these, non-coding RNAs (ncRNAs) have emerged as pivotal modulators of gene expression implicated in vascular inflammation, cell differentiation, lipid handling, and plaque progression [12].

ncRNAs are a diverse class of RNA molecules that do not encode proteins but play fundamental roles in the regulation of gene expression at multiple levels, including epigenetic, transcriptional, and post-transcriptional. Among the most extensively characterized ncRNAs are microRNAs (miRNAs), long non-coding RNAs (lncRNAs), and circular RNAs (circRNAs). miRNAs are short (~20–24 nucleotides) RNA sequences that modulate gene expression by binding partially complementarily to the 3′ untranslated regions (UTR) of target mRNAs, leading to translational repression or degradation. These processes require miRNA association with an Argonaute protein, which is the core component of the RNA-induced silencing complex (RISC) [13]. In contrast, lncRNAs are longer transcripts (>200 nucleotides) that act through a wide variety of mechanisms, interacting with DNA, proteins and other ncRNAs. lncRNAs can act as molecular scaffolds, decoys or guides for chromatin modifiers, thereby influencing transcription, splicing, and epigenetic regulation of neighboring and distant genes [14]. circRNAs, formed through a back-splicing mechanism that generates covalently closed loop structures, are exceptionally stable and often act as miRNA sponges, sequestering specific miRNAs and indirectly modulating gene expression. Additionally, circRNAs can interact with mRNAs or RNA-binding proteins (RBPs), activating or repressing gene expression [15]. Together, these ncRNAs constitute an intricate regulatory network essential for cellular homeostasis. Dysregulation of ncRNA expression has been increasingly implicated in the initiation and progression of carotid atherosclerotic disease.

As the first systematic review devoted exclusively to carotid atherosclerosis, this systematic study aims to provide an unbiased and comprehensive synthesis of the current knowledge regarding ncRNA expression profile, functional roles and regulatory mechanisms across the full spectrum of disease progression—from early, asymptomatic stages to symptomatic cerebrovascular events—thereby providing an unprecedented stage-based molecular scenario of carotid atherosclerosis. Additionally, all included studies were published within the last 15 years, thereby ensuring the contemporary relevance and update potential of this review.

## 2. Methods

### 2.1. Study Design and Search Strategy

We conducted this systematic review following the Preferred Reporting Items for Systematic Reviews and Meta-Analyses (PRISMA) guidelines [16,17]. PRISMA comprises a 27-item checklist to ensure and promote the quality of systematic reviews; this checklist is reported in Supplementary Tables S1 and S2 (for abstract checklist). The protocol employed in the current systematic review has been submitted for registration to the international prospective register for systematic reviews database *Open Science Framework* (OSF) (available online: https://osf.io/x537n; registration DOI: https://doi.org/10.17605/OSF.IO/X537N; accessed on 15 July 2025). The retrieval process consisted of three phases.

### 2.2. Systematic Search Phases

#### 2.2.1. Preliminary Research and Definition of Keywords

During the first phase, started in January 2025, we carried out a preliminary analysis of the literature in the absence of specific keywords to define the scientific databases to be analyzed, the keywords to be used, and the inclusion/exclusion criteria to be applied. Based on the research question and the exploratory research of literature, the strategy was to define keywords referring to the two macro areas of interest: 1. keywords related to Carotid atherosclerosis; 2. keywords related to ncRNA profile. The keywords belonging to each category were summarized in Supplementary Table S3.

#### 2.2.2. Systematic Search and Definition of PICOS

The second phase, conducted in January 2025, consisted of a systematic search among titles, abstracts, and keywords of scientific papers, using the electronic databases PubMed and Scopus, based on the selected keywords, properly combined through the Boolean operators “AND” and “OR”. We limited our search to articles in Italian and English. The search strategy, including all keywords used and the number of studies found from each database, was analytically reported in Supplementary Table S4.

The final selection of papers for inclusion was carried out according to the Population, Intervention, Comparison, Outcomes, and Study Design (PICOS) worksheet [16,17], summarized in Supplementary Table S5. We defined the following PICOS criteria: Population, we chose “Studies in Humans” and “Studies including patients with carotid atherosclerosis” as the inclusion criteria and “In vitro, in vivo studies, and in silico studies” and “Participants with other malignancies or pathologies” as exclusion criteria because we aimed to avoid including humans with carotid atherosclerosis but with any malignancies that might influence the study results, thus preventing the introduction of bias; in addition, we excluded all in vivo, in vitro, and in silico studies. Interventions, we selected as inclusion criterion “Assessment of extracellular, circulating ncRNA expression, and intracellular ncRNAs” in order to include studies that investigated both intracellular and secreted ncRNA differentially expressed in atherosclerosis. Comparisons, we chose “Control Group (Between or Within design)” as an inclusion criterion to enable comparisons between individuals with carotid atherosclerosis and either healthy counterparts or individuals at a different stage of the disease. Outcomes, we chose as inclusion criteria “to provide unbiased and exhaustive overview of the current knowledge on the ncRNAs profile and their mechanism of action in patients with carotid atherosclerosis” and “to summarize the major ncRNA associated with cognitive function in carotid atherosclerosis”, to have a comprehensive understanding of the molecular landscape and its potential implications for cognitive decline in this patient population. Study Design, we chose “Review, Scoping Review, Narrative Review, Systematic Review, Meta-Analysis, Editorial, Book, Case Report, Conference Review, and Conference Paper” as exclusion criteria following the guidelines to carry out a Systematic Review.

#### 2.2.3. Application of PICOS Study Design Exclusion Criteria

The final phase consisted of a first step in which, following PICOS criteria related to the Study Design section, we excluded all reviews, both narrative and systematic ones, meta-analyses, and conference papers, to substantially reduce the number of included studies.

#### 2.2.4. Title and Abstract Selection

By reading the titles and abstract we excluded all studies that did not match with the research question.

#### 2.2.5. Full-Text Selection According to PICOS Criteria

Finally, we included in the systematic review only studies that investigated the ncRNA profile in carotid atherosclerosis. The included papers were read thoroughly to obtain the data of our interest.

#### 2.2.6. Synthesis Method

The included papers were clustered according to the ncRNA profile that may play a role in carotid atherosclerosis and then, for each study, we extracted all the information shown in Tables 1–6 as described next: Study and reference = authors, year of publication; City (Country) = location where the study was conducted. Number of participants = sample size. Source of ncRNA = biological sample from which ncRNAs were extracted and quantified. Comparison = subject groups whose ncRNA expression levels were compared. ncRNA = specific name of the dysregulated non-coding RNA. Dysregulation = indication of whether the ncRNA was found to be upregulated or downregulated in the group of interest. When available, supplementary annotated information includes the ncRNA’s proposed mechanism of action, regulated biological process and plaque features.

### 2.3. Study Risk of Bias Assessment

We assessed the risk of bias for each study in Supplementary Table S6 by compiling the AXIS tool [18].

### 2.4. Network-Level Analysis of Identified ncRNAs

A comprehensive list of proteins implicated in carotid atherosclerosis was compiled by querying two independent databases. First, the GeneCards database was searched using the keyword “carotid atherosclerosis”, and results were filtered to include only “protein-coding” genes. Second, the Molecular Signatures Database (MSigDB) associated with the Gene Set Enrichment Analysis (GSEA) tool was queried using the keyword “atherosclerosis”.

Experimentally validated targets of the miRNAs identified through this systematic investigation were retrieved using the miRWalk online tool, which integrates multiple target prediction algorithms, to provide high-confidence miRNA–mRNA interactions. To ensure reliability, only transcripts with an “NM_” identifier (corresponding to RefSeq mRNA sequences) were retained. The resulting sets of proteins were combined and subsequently intersected with the previously identified proteins associated with carotid atherosclerosis.

Targets that showed a perfect match with carotid atherosclerosis-related proteins were subjected to Gene Ontology (GO) Enrichment Analysis, using the PANTHER Classification System, focusing on the “biological process” category and restricting the analysis to Homo sapiens. Only biological processes with a False Discovery Rate-adjusted *p*-value < 0.05 were considered significant. Among the enriched biological processes, those most closely related to the pathophysiology of carotid atherosclerosis were manually selected and grouped according to their functional relevance. For each identified group, detailed in Supplementary Table S7, miRNA–target interaction networks were constructed and visualized using Cytoscape software (version 3.10.3).

Similarly, to identify the annotated interactors of lncRNAs and circRNAs implicated in carotid atherosclerosis based on this systematic review, the lncTarD 2.0 and circAtlas 3.0 databases were queried. Both direct and indirect targets (predominantly miRNAs and proteins) were recorded. The resulting interaction networks were visualized using Cytoscape. To simplify the network analysis, only protein targets which perfectly matched the previously compiled list of carotid atherosclerosis-associated proteins were retained in the graphical representation.

## 3. Results

### 3.1. Flow Diagram

**Figure 2:**
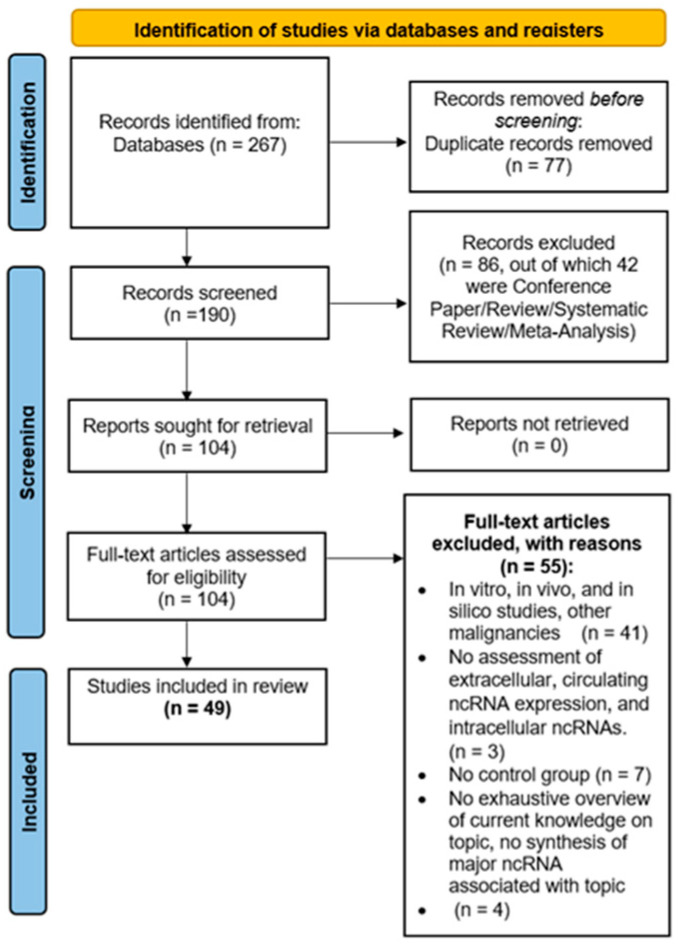
Flow Diagram. The diagram was obtained following the guideline from Page et al. [17].

All the phases in the systematic review process are shown in Figure 2. The research carried out on PubMed and Scopus yielded 80 and 187 studies, respectively. These 267 papers were merged into a non-redundant database and 190 papers remained. Then, by eliminating all studies not related to our research question and all studies that did not match PICOS criteria related to Study Design (exclusion for all types of reviews, meta-analysis, and conference papers), the number of studies was reduced to 104. Finally, by applying all the PICOS criteria, we obtained 49 studies to be included in the systematic review.

### 3.2. Study Selection and Characteristics

Two independent reviewers (G.S. and C.C.) checked the pool of 267 abstracts collected from PubMed and Scopus search engine outputs; any disagreement was discussed with P.B. as the arbiter. Titles and abstracts were screened, and 2922 were removed through a MATLAB standalone application, version R2024a (The MathWorks, Inc., Natick, MA, USA), according to PICOS criteria related to Study Design (42 papers) and irrelevance to the research question (44 papers). The remaining 190 papers were checked for eligibility according to the remaining PICOS criteria, all in vitro, in vivo, and in silico studies and studies with participants with other malignancies (41 papers), all studies without assessment of extracellular, circulating ncRNA expression, and intracellular ncRNAs (3 papers), all studies with no control group (7 papers) and lastly, all studies that did not provide a comprehensive overview of current knowledge on the topic or did not have a summary of major ncRNA associated with topic (4 papers) were excluded. Finally, 49 articles were included, summarized in Table 1, Table 2, Table 3, Table 4, Table 5 and Table 6, which show the main information of each, divided according to the stage of progression of carotid atherosclerosis.

### 3.3. Synthesized Findings

This section outlines the principal findings pertinent to the research objective, as extracted from the 49 studies included in the analysis. All included articles were published within the last 15 years (2010–2024) and originate from research groups across various geographic regions. Specifically, 29 studies were conducted in Asia, with 25 originating from Chinese groups; 11 studies were from Europe, 4 from Russia, 4 from North America (United States and Canada), and 1 from South America. For each publication, significantly dysregulated non-coding RNAs (ncRNAs) are reported based on the statistical thresholds applied in the original studies.

The results are systematically organized into the following subsections, structured hierarchically according to the extent and stage of carotid atherosclerosis within the studied populations. Consequently, ncRNAs identified as dysregulated in multiple comparative contexts may appear in more than one subsection.

All ncRNAs included in this synthesis were identified in human-derived samples, specifically carotid artery tissue, peripheral blood cells, or serum. For each ncRNA, the direction of dysregulation (i.e., upregulation or downregulation) is indicated, along with the proposed molecular mechanism, when reported.

#### 3.3.1. ncRNA Associated with Carotid Atherosclerosis Risk Factors

Although the exact causes or risk factors of carotid atherosclerosis are incompletely determined, certain conditions, traits, or habits may predispose to the development of the disease. The principal recognized risk factors of carotid atherosclerosis are systemic inflammation, high cholesterol and LDL and/or low high-density lipoproteins (HDL) levels in the blood, hypertension, tobacco smoke, diabetes mellitus, obesity, and physical inactivity [68]. The identification of ncRNA signatures associated with these risk factors may offer novel biomarkers for early diagnosis, risk stratification, and preventive intervention. A detailed overview of dysregulated ncRNAs linked to these pathogenic determinants is summarized in Table 1.

The initiation of carotid atherosclerosis involves a series of intricate processes, with inflammatory processes playing a key role in the development and progression of plaque. Adhesion and migration of circulating monocytes through vascular walls, based on the activation of adhesion molecules and chemokines, are key events in the initiation of atherosclerotic plaque [69]. Beside the classical inflammatory biomarkers of carotid atherosclerosis risk prediction—such as proinflammatory cytokines TNF-α and IL-6, high-sensitivity C-reactive protein (hs-CRP) and lipoprotein-associated phospholipase a2 (Lp PLA 2) [70]—Yang and coauthors identified hsa-miR-520b as a novel indicator, significantly downregulated in carotid atherosclerotic plaque versus adjacent non-atherosclerotic tissue [19]. Notably, authors demonstrated that hsa-miR-520b directly interacts with the 3′UTR of NFkB-subunit Rel A transcript, thereby attenuating the expression of adhesion molecules VCAM-1 and ICAM-1 in monocyte and consequently limiting monocyte-endothelial cell adhesion [19].

Metabolic disorders such as diabetes and obesity are characterized by persistent low-grade inflammation, predisposing thus to carotid atherosclerosis and correlated cardiovascular diseases. The primary characteristic trait of diabetics is hyperglycemia, which exerts many cellular effects driving toward endothelial dysfunction and enhanced inflammation [71]. Wang et al. demonstrated that miRNA-29c expression in carotid plaque was decreased in patients with diabetes as compared with the non-diabetic patients, proposing that it could regulate the phenotypic transformation of VSMCs, leading to VSMC proliferation and migration [20]. Obesity may originate from excessive caloric intake along with a sedentary lifestyle, resulting in increased metabolically active subcutaneous adipose tissue, responsible for the secretion of inflammatory adipokines and contributing to atherosclerosis progression [72]. MiRNAs belonging to the let-7 family have been demonstrated to exert an atheroprotective function, by modulating VSMC and endothelial cell activation and inflammation, and suppressing vascular inflammation mediators such as interleukin (IL)-6, IL-1β and NFkB [73,74]. Elevated serum levels of let-7d-5p have been detected in overweight patients with carotid atherosclerosis relative to lean counterparts and healthy controls, although this upregulation is absent in obese atherosclerotic individuals, possibly due to advanced systemic inflammation impairing let-7d-5p homeostasis [21].

Hypercholesterolemia, particularly elevated LDL-cholesterol (LDL-C), remains the most important risk factor for carotid atherosclerosis. Mandolini et al. reported increased hsa-miR-33b and hsa-miR-758, in carotid plaques from hypercholesterolemic patients (LDL-C > 4.14 mmol/L), compared to the normocholesterolemic carotid atheroma [22], while Tanashyan et al. found both strands of hsa-miR-33a upregulated in blood of patients with an LDL-C ≥ 1.8 mmol/L presenting carotid plaque [23]. Interestingly, these miRNAs directly target ATP Binding Cassette Subfamily A Member 1 (ABCA1), required for cholesterol efflux and, thus, essential for maintaining cellular cholesterol homeostasis [75,76].

In accordance with the recommendations of the American College of Cardiology/American Heart Association [77] and with the European Society of Hypertension and European Society of Cardiology [78], essential hypertension is defined as diastolic blood pressure ≥ 90 mmHg or systolic blood pressure ≥ 140 mmHg, or the use of antihypertensive medications. Hypertension damages endothelium by increasing the hemodynamic pressure on endothelium and may increase the permeability of arterial walls for lipoproteins. Zhang et al. identified five circRNAs upregulated in hypertensive individuals with carotid atherosclerosis relative to normotensive controls [24]. Conversely, Qian et al. described three circRNAs with reduced expression in hypertensive patients with carotid plaques, among which circ-0127342 was specifically diminished in hypertensives with plaques versus those without, suggesting its potential as a disease-specific biomarker [25].

#### 3.3.2. ncRNA Correlated with Carotid Intima-Media Thickness (cIMT)

In the primary prevention setting, the presence of increased cIMT is validated as a marker of subclinical atherosclerosis and, as such, a predictor of carotid plaque formation and atherosclerosis [79]. Thus, identification of ncRNAs modulated in early atherogenesis is pivotal for timely intervention. Relevant biomarkers discovered in the present systematic review are presented in Table 2. Minin and collaborators found significantly higher expressions of hsa-miR-145-5p and hsa-miR-let7c in the serum of patients with carotid plaques relative to those with increased cIMT alone, suggesting that these miRNAs could be used for distinguishing the early phase from a more advanced stage of carotid atherosclerosis [26]. A subsequent study demonstrated the significantly higher expression of lncRNA SOX2-OT in serum of subjects with increased cIMT compared to healthy individuals, which was proposed as a valuable clinical marker of atherosclerosis, with diagnostic metrics including an AUC of 0.921, sensitivity of 87.4%, and specificity of 82.2% [27]. Yan et al. described a progressive upregulation of hsa_circ_0043621 in serum from healthy individuals to those with cIMT and ultimately with plaques, suggesting a linear correlation with disease severity [28]. Notably, all these studies defined increased cIMT as 1.0 < cIMT < 1.5 mm, while the presence of plaque is indicated by a cIMT value higher than 1.5 mm or a focal structure that encroached into the arterial lumen measuring at least 0.5 mm or 50% of the surrounding cIMT [26,28].

#### 3.3.3. Differentially Expressed ncRNA in Carotid Atherosclerosis Versus Healthy Controls

From this study, several classes of ncRNA have resulted differentially expressed in individuals with carotid atherosclerosis with respect to non-atherosclerotic subjects, and summarized in Table 3, with miRNAs representing the predominant class. Among the upregulated miRNA in carotid atherosclerosis patients compared to healthy control, hsa-miR-33a and hsa-miR-33b had been identified by three distinct studies, both in serum and in carotid tissue [30,33,35]. Notably, this subgroup of miRNA directly targets 3′ UTR of ABCA1 transcript, thus regulating the cholesterol uptake. hsa-miR-148a-3p, another direct regulator of ABCA1 expression, was found upregulated in plasma of carotid stenosis patients [30]. In contrast, miRNA-148b expression resulted significantly lower in carotid atherosclerosis [40]. Conflicting evidence surrounds hsa-miR-21; two studies reported its upregulation [31,36], whereas a third study observed a lower expression of both the hsa-miR-21-5p and the -3p strand [35]. Additional upregulated circulating miRNAs in carotid atherosclerosis group included hsa-miR-146a [29], let-7d-5p [21], hsa-miR-200c [33], hsa-miR-135a, hsa-miR-137, hsa-miR-149, and hsa-miR-219a [34]. Higher expression of hsa-miR-127-3p [32], hsa-miR-19b, hsa-miR-22 and hsa-miR-143 [36] was found in carotid tissue from atherosclerotic patients when compared to non-atherosclerotic subjects.

Conversely, several miRNAs were consistently downregulated in carotid atherosclerosis. Two different studies reported a significative reduction in serum level of hsa-miR-126 in carotid atherosclerosis patients [34,35], which has been demonstrated to directly target cycloxigenase-2 (COX2) 3′ UTR. Significantly lower levels of hsa-miR-320b [37], hsa-miR-638 [38], hsa-miR-145 [39], hsa-miR-216b [44], hsa-miR-223, hsa-miR-101, hsa-miR-577 and hsa-miR-384 [34] were found in serum of atherosclerotic patients with respect to healthy control. Additionally, downregulated expression levels of hsa-miR-210 [41], hsa-miR-1, hsa-miR-29b and let-7f [36] were found in carotid plaque compared to normal carotid tissue.

Several lncRNAs exhibited altered expression in carotid atherosclerosis, thus representing valid biomarkers for diagnosis. Circulating lncRNA AC078850.1 was upregulated and shown to interact with Hypoxia Inducible Factor 1 (HIF-1) transcription factor, forming a ribonucleoprotein which promotes ROS production, Nod-like receptor protein 3 (NLRP3) inflammasome activation and foam cell formation in macrophages [42]. Similarly, the lncRNA MIAT, involved in the promotion of inflammation in macrophages and plaque progression, was upregulated in carotid plaque compared with the normal control artery [43]. Another lncRNA was found to be upregulated both in serum and in carotid plaque from atherosclerotic patients and associated with VSMCs migration and proliferation is LINC01123 [45]. Mechanistically, LINC01123 directly binds and sequesters hsa-miR-1277-5p, which is responsible for regulating the expression of transcription factor Krueppel-like factor 5 (KLF5) [45]. Consistently, authors also found that the expression level of hsa-miR-1277-5p itself was downregulated in serum form in affected individuals [45]. Lou et al. reported the downregulation of SENCR, alongside the reciprocal upregulation of its target hsa-miR-126a, in carotid plaque with respect to healthy arteries [47]. Similarly, downregulation of lncRNA FGF7-5 and lncRNA GLRX3, and the concomitant upregulation of its target miRNA, hsa-miR-2681-5p, was observed in serum of atherosclerotic patients compared to those from healthy volunteers [48]. Additional dysregulated lncRNAs in carotid atherosclerosis subjects resulting from this research included CCAT2 [44] and HOXC-AS1 [46].

Finally, Yan et al. identified three circRNA, specifically circ-0043621, circ-0051995 and circ-123388, upregulated in peripheral blood mononuclear cells (PBMC) from carotid atherosclerosis patients with respect to healthy subjects [28]. Among them, circ-0043621 was shown to directly bind hsa-miR-223-3p and negatively regulate its expression. Thus, authors found that hsa-miR-223-3p expression was significantly downregulated [28], corroborating earlier findings of reduced hsa-miR-223-3p levels in carotid atherosclerosis [34].

#### 3.3.4. ncRNA and Carotid Plaque Features

Atherosclerotic involvement of the carotid artery displays considerable histopathological and biological heterogeneity. Carotid plaques do not exhibit uniform structural or molecular characteristics, and such differences are largely governed by distinct epigenetic mechanisms, primarily mediated by ncRNAs. Table 4 provides a comprehensive overview of ncRNAs exhibiting differential expression profiles associated with specific plaque phenotypes identified through this systematic review.

The plaque core markedly differs from the adjacent proximal region. In addition to the accumulated atheromatous material, the plaque zone is enriched in macrophages compared to the adjacent region, which is predominantly composed of VSMCs [49]. Accordingly, these two distinct regions exhibit unique ncRNA expression profiles. Raju and collaborators identified a group of upregulated miRNAs in plaque—namely hsa-miR-146a, hsa-miR-155, let-7a, hsa-miR-200b, hsa-miR-223 and hsa-miR-181b—with respect to the corresponding marginal zone, from macrophage-derived extracellular vesicles [49]. In contrast, Yan et al. reported a lower expression level of hsa-miR-223 in plaque relative to surrounding tissue, where its expression is modulated by circ-0043621 [28]. Indeed, the latter is found upregulated in plaque region with respect to the marginal zone, positively regulating its downstream target NLRP3, required for atherogenesis [28]. Another upregulated marker in carotid plaques compared to the common carotid region is hsa-miR-148a-3p, whose function is to negatively regulate the ABCA1 expression, a key transporter involved in cholesterol efflux [30].

Carotid plaques present histological and phenotypical differences which ultimately influence clinical outcomes. One key anatomical parameter is the degree of stenosis, angiographically quantified as:

% Stenosis = (1 − A_stenosis_/A_open_) × 100

where A_stenosis_ and A_open_ are the cross-sectional areas of the stenosis throat and open lumen, respectively [80].

According to the North America Symptomatic Carotid Endarterectomy Trial (NASCET) criteria, stenosis is classified as mild (<30%), moderate (30–69%), or severe (70–99%), with 100% indicating complete occlusion [81]. Huang et al. reported progressive increase in hsa-miR-146a in PBMC from patients with increasing stenosis severity [29]. Another research group found that patients with advanced stenosis exhibited decreased levels of circulating hsa-miR-126-5p and -3p, hsa-miR-21-5p and -3p and hsa-miR-29a-3p, along with elevated levels of hsa-miR-33a (both -5p and -3p strands) compared to subjects with moderate carotid stenosis [50]. Stenosis progression, defined as the temporal increase in arterial narrowing due to plaque growth, is associated with heightened risk of ischemic events [82]. Dolz et al. analyzed serum miRNA profiles in patients over a two-year follow-up and identified increased expression of hsa-miR-199b-3p, hsa-miR-130a-3p, and hsa-miR-24-3p in individuals exhibiting stenosis progression, suggesting their potential role as prognostic biomarkers [51].

From a clinical perspective, plaques are often classified as “stable” or “unstable,” reflecting their rupture potential. Stable plaques exhibit low rupture risk, whereas unstable (or ruptured) plaques are prone to embolization, increasing the likelihood of cerebrovascular events [83]. These phenotypes are mainly due to their biochemical composition. Histological analyses distinguish vulnerable from stable plaques based on structural features; vulnerable plaques possess large lipid-rich cores, reduced calcification, and thin fibrous caps (<200 µm), whereas stable plaques exhibit higher calcific content, denser fibrous tissue, and thicker caps (>200 µm), conferring mechanical stability [84,85].

Identifying vulnerable plaques before they become unstable is critical for preventing major cardiovascular events. Numerous miRNAs have been associated with plaque vulnerability, which may serve as potential biomarkers. Wang and collaborators examined the expression level of hsa-miR-124 in serum of patients with acute cerebral infraction, demonstrating a significantly higher expression in patients with vulnerable carotid plaque compared to the those with stable lesions [52]. Similarly, Huang et al. reported an increased PBMC expression of hsa-miR-146a in vulnerable plaque patients [29]. Yang et al. found a panel of upregulated circulating miRNAs—specifically hsa-miR-23a-5p, hsa-miR-320a, hsa-miR-2110 and hsa-miR-134-5p—in vulnerable versus stable plaque, while also noting lower expression of hsa-miR-4439 in the vulnerable group [53]. Other miRNAs downregulated in subjects with vulnerable carotid plaque include hsa-miR-532-3p [54] and hsa-miR-320b [37].

Regarding calcification, although controversial, the prevailing hypothesis suggests that highly calcified plaques are generally more stable and less symptomatic than non-calcified ones of similar size [86]. Katano et al. found that hsa-miR-4530, hsa-miR-133b and hsa-miR-1-3p were more highly expressed in low-calcified plaques, with respect to high-calcified plaques with a similar degree of stenosis [56]. Vasuri et al. compared calcification subtypes and reported significantly higher expression of hsa-miR-30a-5p and hsa-miR-30d in protruding nodular calcifications versus calcific cores [55].

Furthermore, molecular profiling of carotid plaques might predict the progression of the pathology and help identify patients at high risk of cerebral events, who could benefit from earlier surgical revascularization. Numerous studies investigated the different expression levels of ncRNAs by comparing stable and unstable plaques. Reportedly, unstable plaques exhibited higher level of hsa-miR-200c [33], hsa-miR-127-3p [32] and hsa-miR-330-5p [57] with respect to stable plaque. Conversely, Eken et al. observed decreased levels of hsa-miR-210 and hsa-miR-21 in ruptured plaque when compared to stable plaque [41], while Zhu and coworkers identified lower serum level of hsa-miR-126 and hsa-miR-223 in patients with unstable carotid plaque with respect to subject with stable lesion [34]. Badacz et al. analyzed miRNA profiles in different plaque groups, based on plaque echogenicity. Plaque echogenicity is an important characteristic used to assess plaque composition and stability, which refers to the ability of an atherosclerotic plaque to reflect ultrasound waves during imaging, such as carotid or intravascular ultrasound. Patients with hyperechogenic plaque showed significantly higher serum levels of hsa-miR-134-5p, hsa-miR-34a-5p and hsa-miR-375 when compared to patients with moderately echogenic carotid plaque. Interestingly, these miRNAs were further reduced in the hypoechogenic group compared to the moderately echogenic one, suggesting a progressive downregulation of these markers with the decrease in echogenicity [58]. Conversely, the serum level of hsa-miR-133b was higher in the hypoechogenic plaque group with respect to the moderately echogenic group [58]. Notably, hsa-miR-16-5p was the only miRNA significantly upregulated in hyperechogenic versus hypoechogenic groups [58]. Additionally, hsa-miR-1-3p and hsa-miR-16-5p were both elevated in patients with ulcerated plaques [58].

Beyond miRNAs, other ncRNA classes—particularly long non-coding RNAs (lncRNAs) and circular RNAs (circRNAs)—also show distinct expression patterns between stable and unstable plaques. MIAT and CCAT2 lncRNAs, previously mentioned as upregulated in carotid plaque group with respect to healthy control, were further elevated in unstable plaques [43,44]. Bao et al. identified 488 lncRNAs and 91 circRNAs differentially expressed in stable versus unstable plaques. Among them, ENST00000430222, ENST00000602895, circ-013041 and circ-025902 were upregulated in the unstable group, while ENST00000631338, MSTRG18183, circ-054182 and circ-037511 were downregulated [59]. Finally, unstable plaque was characterized by a higher level of circulating circRNA-0006896 [60], as well as of circ-0001523, circ-0008950 and circ-0000571, but by reduced expression of circ-0001946 and circ-0000745, compared to stable plaque [61].

#### 3.3.5. Symptomatic vs. Asymptomatic Patients

As previously stated, carotid plaques may remain stable and asymptomatic for extended periods or may gradually worsen and evolve into unstable lesion. Unstable plaques can release atheromatous material or thrombi into the cerebral circulation, leading to clinical manifestations such as TIA, transient monocular vision loss ipsilateral to the affected artery (amaurosis fugax), or minor/non-disabling ipsilateral strokes [30]. The evaluation of ncRNAs expression profile in symptomatic versus asymptomatic patients offers valuable insights into the molecular pathways underlying symptom onset and provides potential prognostic biomarkers for cerebrovascular risk, revealing a wide array of dysregulated miRNAs distinguishing symptomatic from asymptomatic individuals (Table 5).

Recently, Tanashyan et al. analyzed the expression of a total of 24 miRNAs in plasma from carotid atherosclerosis patients, reporting a significant upregulation of hsa-miR-200c-3p, hsa-miR-106b-5p and hsa-miR-494-5p in symptomatic subjects, alongside a marked reduction in hsa-miR-183-3p, hsa-miR-126-5p and hsa-miR-216-3p [63]. In a microarray-based study assessing 742 miRNAs, Eken et al. identified hsa-miR-210 as the sole miRNA significantly downregulated in symptomatic patients [41]. Mechanistically, authors proposed hsa-miR-210 as a direct negative regulator of Adenomatous Polyposis Coli (APC) protein, affecting the canonical Wnt signaling in VSMCs and promoting atherosclerosis progression. Moreover, another study documented a significative increase in hsa-miR-29c expression in carotid artery of symptomatic patients undergoing carotid endarterectomy (CEA) [20]. Interestingly, this finding was corroborated in specimens from stroke patients (see following subsection, [20]). Badacz et al. analyzed organ-specific circulating microRNAs—namely cardiac, skeletal muscle, brain, liver and pancreas related miRNAs—in serum from symptomatic and asymptomatic subjects, reporting a significative upregulation of brain-derived miRNAs in symptomatic subjects, specifically hsa-miR-124-3p and hsa-miR-134-5p, both inversely correlated with plaque stability [58]. Grosse and colleagues identified hsa-miR-92a as the only miRNA significantly increased in plasma from symptomatic compared to asymptomatic individuals [62]. Other upregulated miRNAs in symptomatic patients are hsa-miR-148a-3p and hsa-miR-33a-5p [30], with the latter also being upregulated in patients with high-grade stenosis [50], likely suggesting a possible positive correlation between hsa-miR-33a-5p expression and carotid atherosclerosis severity. Several others reported miRNAs were found to be downregulated in carotid tissue from symptomatic patients, including hsa-miR-21, hsa-miR-143 [36], hsa-miR-484, hsa-miR-942 and hsa-miR-1287 [64].

#### 3.3.6. Association of ncRNAs with Ischemic Stroke

Stroke remains a massive public health problem with an increasing need for better strategies to prevent and treat disease in our aging society. Although some ischemic events are caused by reduced blood flow (hypoperfusion), the majority are attributable to embolic mechanisms linked to atherosclerotic plaque rupture or acute carotid occlusion, leading to downstream thrombus migration to distal regions of the brain [87].

The expression profiles of ncRNAs can vary substantially during cerebrovascular disease. Table 6 outlines the dysregulated ncRNAs in individuals with carotid atherosclerosis-associated cerebrovascular events, emerging from this systematic research. Recently, authors investigated the expression of circulating ncRNAs in patients with carotid artery- related atherosclerotic cerebrovascular events. Particularly, a total of 225 patients were classified into low-risk, medium-high risk of cerebrovascular event groups and control group which included healthy subjects without carotid plaque. They reported elevated hsa-miR-874 levels in the medium-high risk group, while circSCMH1—a circular RNA implicated in neural repair and known to interact with hsa-miR-874 [67,88]—was significantly downregulated in the same group [67,88].

In a separate study involving 167 stroke patients and 157 healthy subjects, researchers observed higher serum levels of hsa-miR-21 and decreased levels of hsa-miR-221 in the stroke cohort [31]. Interestingly, the downregulation of hsa-miR-221 was independently confirmed by two additional studies. Specifically, Bazan et al. observed reduced levels of hsa-miR-221 and hsa-miR-222 in carotid tissues from “urgent” patients—those undergoing CEA within five days of a neurological event—compared to asymptomatic or symptomatic individuals [65]. A subsequent study by the same group demonstrated the downregulation of hsa-miR-221 in a different cohort of urgent subjects with carotid atherosclerosis, also demonstrating a negative regulation operated by circR-284 [66]. Consistently, the expression level of circR-284 was higher in serum from urgent subjects with carotid atherosclerosis [66]. Wang and colleagues studied the expression level of has-miR-29c in a cohort of carotid plaque specimens collected from patients with or without cerebral events, reporting an upregulation of hsa-miR-29c in the cerebral stroke group compared with the non-cerebral stroke group [20]. Lastly, Luque and collaborators identified a downregulation of hsa-miR-638 in serum from atherosclerosis stroke patients versus non- atherosclerotic controls, proposing it as a potential biomarker for primary and secondary ischemic stroke risk prediction [38].

#### 3.3.7. Interaction Network Analysis

##### miRNA-Target Interaction Network

Since the measurements of ncRNAs were not obtained using quantitative methods in the studies included in this systematic review, the comparison of data by meta-analyses was not performed. Therefore, to improve the interpretation of the results without changing the methodology and nature of this systematic review, a functional analysis of identified ncRNAs was performed by interaction network analysis. Uncovering the molecular mechanisms underlying carotid atherosclerosis requires the integration of regulatory and functional data. In this context, miRNA–target interaction networks represent a powerful approach to reveal how post-transcriptional regulation may influence key pathogenic pathways.

Thus, we focused specifically on the 51 circulating miRNAs associated with carotid atherosclerosis, resulting from the analysis of included articles. To prioritize miRNAs with functional relevance to carotid atherosclerosis, we matched their validated targets (via the miRWalk database) with a curated list of protein-coding genes known to be associated with carotid atherosclerosis, obtained from GeneCards and MSigDB. This intersection led to the identification of 13 miRNAs with high-confidence interactions with atherosclerosis-related genes. These miRNAs were subjected to Gene Ontology enrichment analysis and network-based visualization to explore their regulatory roles.

**Figure 3:**
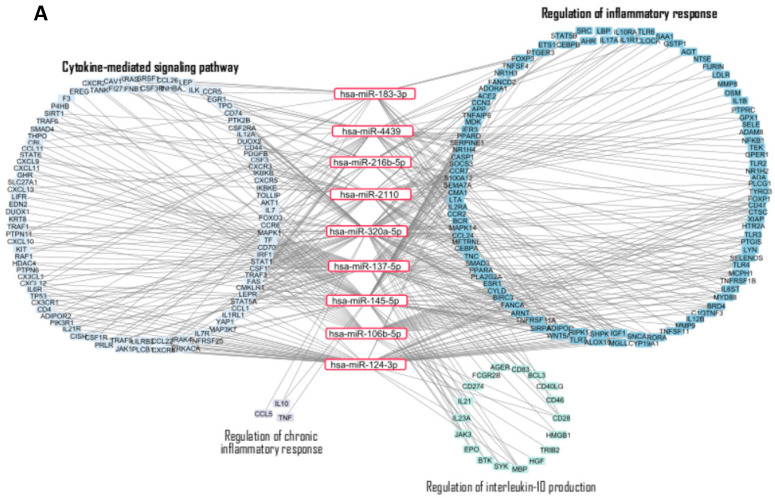

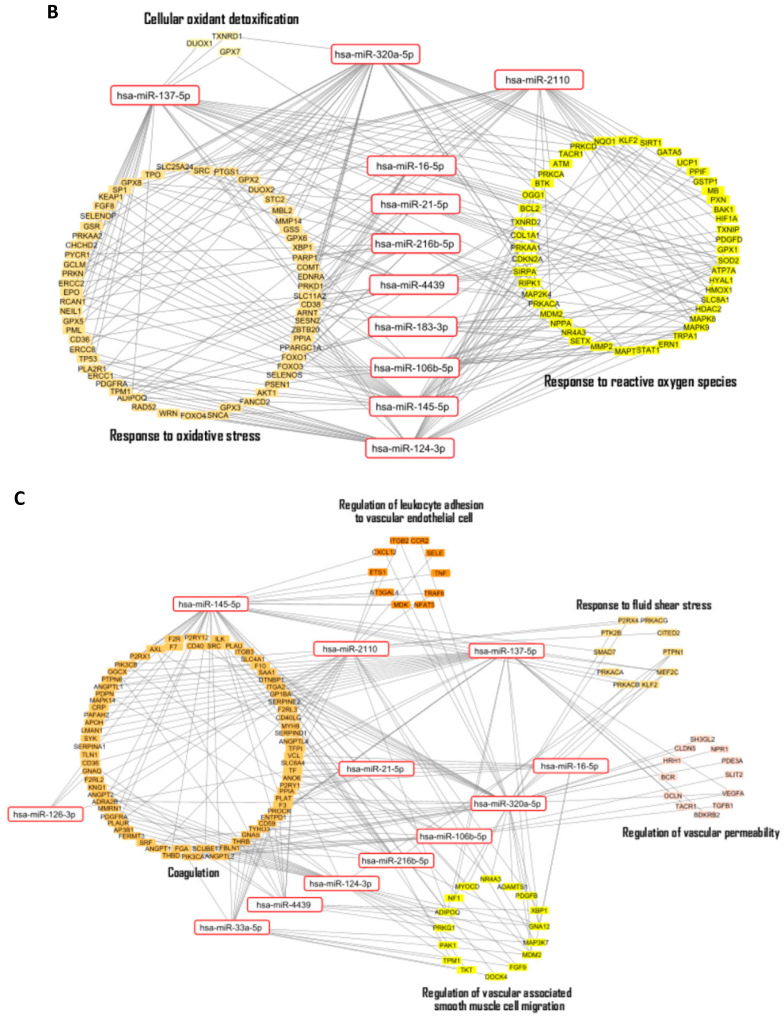

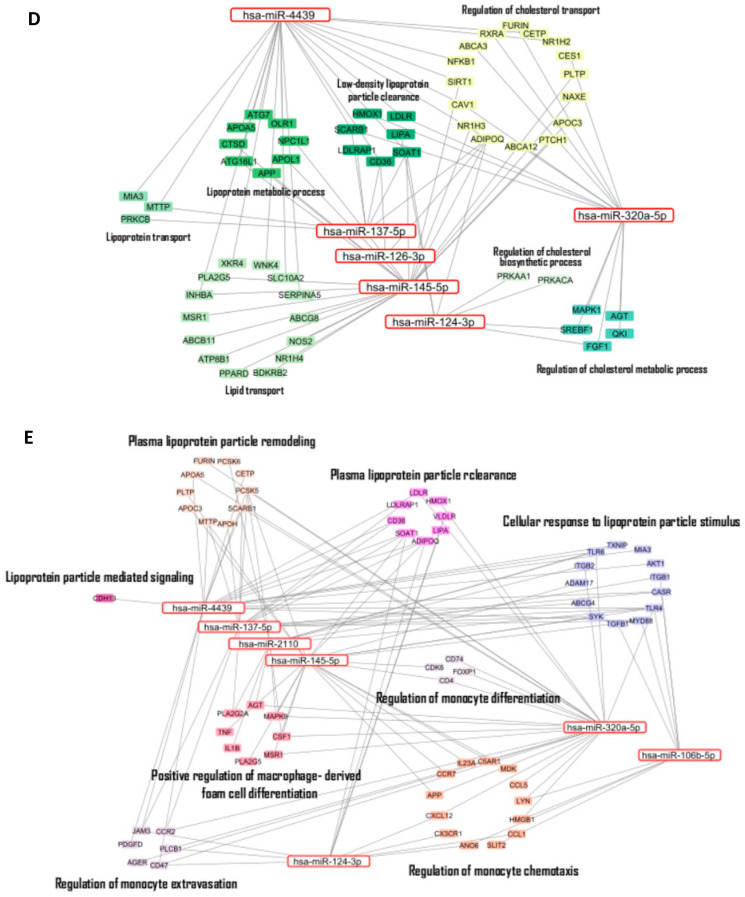
miRNA–target interaction networks involved in carotid atherosclerosis. (**A**). Network of miRNA–target interactions involved in inflammation and immune response. (**B**). Network of miRNA–target interactions involved in oxidative stress. (**C**). Network of miRNA–target interactions involved in endothelial and vascular response. (**D**). Network of miRNA–target interactions involved in lipid metabolism. (**E**). Network of miRNA–target interactions involved in plaque formation and foam cells. Red rectangles represent miRNAs, while colored rectangles indicate their target genes, grouped by the associated biological process. The edges between nodes represent regulatory interactions. The graphs were generated in Cytoscape 3.10.3 and visualized with the Group Attributes Layout.

Five interaction networks were generated using Cytoscape, each corresponding to a distinct biological area involved in atherogenesis: inflammation, oxidative stress, vascular response, lipid metabolism and plaque formation. Each network revealed both hub miRNAs with broad targeting capabilities and more specialized miRNAs with selective pathway involvement (Figure 3).

The network focused on inflammation and immune response. (Figure 3A) illustrates the complex interactions between selected miRNAs and their target genes involved in inflammatory signaling. hsa-miR-145-5p, hsa-miR-320a-5p, and hsa-miR-137-5p emerged as key regulators, showing high connectivity with pro-inflammatory targets. Notably, miR-124-3p and miR-106b-5p are linked to multiple genes involved in interleukin-10 regulation, suggesting their potential contribution to the resolution of inflammation.

The second network (Figure 3B) captures miRNA–target interactions related to oxidative stress and cellular responses to reactive oxygen species. Several miRNAs, including hsa-miR-124-3p, hsa-miR-320a-5p, and hsa-miR-145-5p, exhibit multiple connections with genes involved in antioxidant defense and redox homeostasis. For example, hsa-miR-320a-5p targets are clustered around detoxification processes related to glutathione metabolism, whereas hsa-miR-145-5p appears to regulate transcription factors activated by oxidative stimuli, highlighting their potential role in preserving redox balance within the vascular microenvironment.

Figure 3C shows miRNA–target interactions involved in endothelial function and vascular response. According to our analysis, hsa-miR-137-5p, hsa-miR-320a-5p and hsa-miR-2110 exhibit broad targeting profiles, whilst hsa-miR-126-3p and hsa-miR-21-5p are more specifically linked to genes involved in coagulation.

The fourth network (Figure 3D) reveals six miRNAs involved in key lipid-metabolism pathways. Among them, hsa-miR-4439 appears as a central node, targeting multiple genes involved in lipoprotein metabolism and cholesterol transport, suggesting its potential role in modulating plasma lipid levels and lipid deposition within the arterial wall.

Lastly, we analyzed miRNA–target interactions involved in foam cell differentiation, cholesterol accumulation, and plaque development. This network (Figure 3E) is enriched in genes related to cholesterol efflux, lipid transport, and macrophage-derived foam cell formation, with seven miRNAs participating in this regulatory scenario. Notably, hsa-miR-320a-5p stands out for its involvement across almost all identified biological processes, indicating a prominent regulatory role in atherogenesis.

##### lncRNA and circRNA–Target Interaction Network

The analysis of the targets of other ncRNAs (namely lncRNAs and circRNAs) proved to be more challenging, resulting in a less comprehensive and more approximate network due to the limited availability of target annotations for these two classes of ncRNAs. Firstly, the databases used—lncTarD 2.0 for lncRNAs and circAtlas 3.0 for circRNAs—did not provide experimentally validated targets but rather computationally predicted interactions generated by specific algorithms. Secondly, many of the molecules identified through the systematic search were not found in these databases. In fact, among the 13 lncRNAs identified, targets could be retrieved for only 8, whereas among the 21 circRNAs detected, predicted targets were available for only 5 of them.

**Figure 4:**
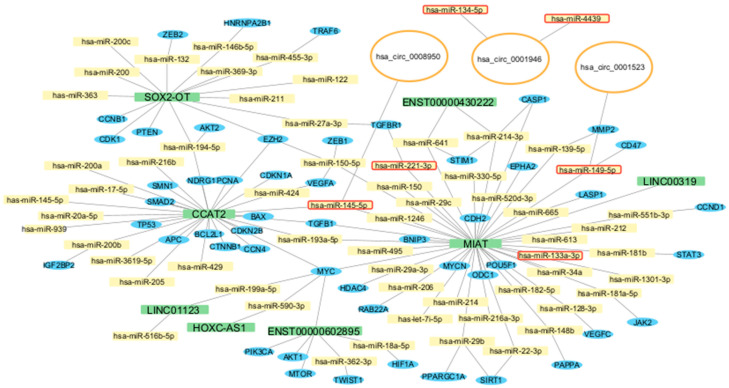
Interaction networks between lncRNA and circRNA involved in carotid atherosclerosis and their targets. The interaction analysis was performed for the lncRNAs and circRNAs identified through the systematic search, as described in the Methods section. lncRNAs are represented as green bars, while circRNAs are shown as orange empty ellipses. miRNA targets are indicated by yellow bars, and protein-coding targets are represented as blue circles. miRNAs outlined in red correspond to those previously reported in the literature as dysregulated in atherosclerotic patients, supporting the biological relevance of these interactions. The graph is generated in Cytoscape 3.10.3.

Figure 4 shows the analyzed lncRNAs together with the three most relevant circRNAs (hsa_circ_0008950, hsa_circ_0001946, and hsa_circ_0001523), which appeared to act as molecular sponges for miRNAs that were also independently retrieved through the systematic search, thereby reinforcing the consistency of the network with the bioinformatic screening. Among these, hsa-miR-4439 was a particularly prominent marker, also identified in the previous miRNA–target network analysis and implicated in all five functional areas considered. Another miRNA, hsa-miR-145-5p, was found to interact with one circRNA and two distinct lncRNAs. In the previous analysis, this miRNA was associated with processes related to inflammation and immune response, oxidative stress, endothelial and vascular regulation, as well as plaque formation and foam cell development.

Regarding lncRNAs, MIAT emerged as a central hub, showing extensive connections with numerous miRNAs (e.g., hsa-miR-133a-3p, hsa-miR-149-5p, hsa-miR-145-5p, already mentioned among the circulating miRNAs identified from the reviewed articles) and several downstream protein-coding targets, including CDH2, VEGFC, and STAT3, which are known to play roles in endothelial function, angiogenesis, and inflammatory signaling. Similarly, CCAT2, SOX2-OT, and ENST00000602895 exhibited relevant interactions with key regulatory miRNAs and downstream targets such as MTOR, AKT1, HIF1A, PIK3CA, CTNNB1, and VEGFA, suggesting their potential contribution to vascular remodeling and cell proliferation pathways.

Overall, this integrative analysis supports the hypothesis that the interplay between lncRNAs, circRNAs, and miRNAs may coordinate multiple pathogenic mechanisms underlying carotid atherosclerosis, including vascular inflammation, endothelial dysfunction, and smooth muscle cell proliferation.

## 4. Discussion

Carotid atherosclerosis remains undoubtedly the most important contributor to ischemic stroke, exerting a substantial global burden in terms of neurological disability and mortality among adults. Atherosclerotic plaque formation and progression is an irreversible, multistep process, regulated by a complex interplay of molecular and cellular factors. As the first systematic review exclusively focused on carotid atherosclerosis, this study integrates recent (post-2010) findings on ncRNA differential expression, systematically classified by disease stage—from subclinical atherosclerosis to overt ischemic stroke—and across different biological samples (including serum, PBMCs and carotid tissue). Importantly, given that this review specifically addresses carotid atherosclerosis, while certain ncRNA-related mechanisms may also operate in coronary or other vascular beds, direct extrapolation should be interpreted with caution. Differences in hemodynamics, tissue-specific gene expression, and the local microenvironment between carotid and coronary arteries may influence ncRNA expression and function, precluding the generalizability of these findings to other atherosclerotic districts.

This research highlights the potential of ncRNAs as molecular biomarkers and therapeutic targets, identifying three classes of dysregulated ncRNAs: circRNA, lncRNa and, most frequently, miRNA. Many of these molecules are functionally involved in key atherogenic processes such as inflammation, VSMC phenotypic switching, and ABCA1-mediated cholesterol efflux—mechanisms fundamental to plaque initiation, progression, and destabilization.

The let-7 family, known for its anti-inflammatory and atheroprotective functions, emerged positively associated with obesity, increased cIMT and presence of carotid plaque. Among the most frequently dysregulated miRNAs hsa-miR-146a and hsa-miR-221 drive regulatory shift promoting atherosclerotic lesion development and symptomatology. Conversely, hsa-miR-126 and hsa-miR-210 were consistently reduced with disease progression, reflecting their protective roles in repressing COX2 expression and maintaining VSMC stability. The miR-33 and miR-148 families emerged as key regulators of LDL-cholesterol homeostasis via direct repression of the ABCA1 transporter, correlating with hypercholesterolemia, plaque presence, and symptomatic stenosis. Similarly, the miR-29 family, which modulates macrophage polarization and inflammation, showed stage-dependent expression patterns—suggesting a dual role in plaque stabilization and destabilization.

Finally, network analysis revealed complex and multifactorial roles of these molecules in the molecular landscape of carotid atherosclerosis, with 13 circulating miRNAs with validated atherosclerosis-related targets (including hsa-miR-16-5p, hsa-miR-21-5p, hsa-miR-33a-5p, hsa-miR-126-3p, hsa-miR-145-5p, and hsa-miR-4439), alongside 8 lncRNAs and 5 circRNAs with predicted involvement. The stability and detectability of circulating ncRNAs in minimally invasive samples underscore their promise as diagnostic and therapeutic tools.

## 5. Limitations

Besides the extensive exploration of the literature on ncRNAs in carotid atherosclerosis, some limitations should be acknowledged. Quantitative methodologies for absolute measurement of ncRNA were not employed in the included studies—most of which relied on relative quantification via Real Time PCR, potentially limiting cross-study comparability—and meta-analysis could not be performed. Absolute quantification of distinct ncRNA species, for example, through digital PCR, could enable more robust comparisons of expression levels across independent cohorts. Additionally, it would allow the definition of clinically relevant thresholds for disease stratification or prognosis and support the translation of ncRNA biomarkers into clinical practice.

Moreover, the majority of the studies included in this review focused on miRNAs, while lncRNA and circRNAs are far less explored. Due to their more complex and multifaceted mechanisms of action compared to miRNAs, many of these molecules remain incompletely annotated, and their biological functions are poorly understood. In addition, their often-low expression levels, tissue- and stage-specificity, and variable stability further complicate reliable detection and quantification. Collectively, these factors contribute to incomplete annotation in public databases, which also limited the analysis of circRNA–target interaction networks in the present review; indeed, only 5 out of the 22 identified circRNAs had annotated target information available in public resources, preventing conclusive network analyses.

An additional limitation concerns the geographical distribution of the selected studies; approximately half were conducted in Chinese cohorts. This may restrict the generalizability of the findings to other populations, as genetic background, environmental factors, lifestyle, and comorbidities can influence ncRNA expression and their association with carotid atherosclerosis. Validation studies in diverse ethnic and geographic cohorts are therefore necessary to determine whether the observed ncRNA patterns and potential biomarker roles are globally applicable.

## 6. Conclusions

This systematic review provides an updated and comprehensive synthesis of the last fifteen years of research on ncRNAs in individuals with carotid atherosclerosis, offering novel insights into their regulatory roles throughout the natural history of the disease.

Collectively, these findings underscore the multifactorial and stage-dependent involvement of ncRNAs in the pathobiology of carotid atherosclerosis, from early endothelial dysfunction and plaque formation to symptomatic events and ischemic stroke. For the first time, the dynamic expression pattern of different ncRNA classes is delineated along the progression of carotid atherosclerosis, highlighting their potential as biomarkers and modulators of vascular pathology and stroke.

However, most of the reviewed studies were conducted on limited patient cohorts, highlighting the urgent need for large-scale, longitudinal investigations to validate and translate these molecular insights into clinical applications. On the other hand, no clinical trials are currently available that evaluate ncRNAs as biomarkers of atherosclerosis in clinical settings [89], but only synthetic ncRNAs—such as small interfering RNAs (siRNAs)—are being investigated as therapeutic strategies to counteract disease progression [90]. Finally, harmonizing nsRNA detection using quantitative methods will ensure the reliability and reproducibility of findings and comparisons across studies, thus allowing the implementation of ncRNAs in clinical practice.

## Figures and Tables

**Table 1:** ncRNA associated with carotid atherosclerosis risk factors.

Study and Reference	City and Country	Number of Participants	ncRNA	Dysregulation	Source of ncRNA	Proposed Mechanism
***Endothelial inflammation***						
Yang et al. 2021 [19]	Beijing (China)	N = 3 control N = 3 CA	hsa-miR-520b	downregulated in CA	tissue	Direct interaction of hsa-miR-520b and RelA transcript
***Diabetes mellitus (DM)***						
Wang et al. 2024 [20]	Luoyang (China)	N = 25 DM N = 15 non-DM	hsa-miR-29c	Downregulated in DM patients	tissue	VSMC phenotype switching
***Obesity***						
Aroca-Esteban et al. 2024 [21]	Madrid (Spain)	N = 7 control N = 4 lean CA N = 10 overweight CA N = 5 obese CA	let-7d-5p	upregulated in overweight (but not obese)	plasma (extracellular vesicle)	protective role in the inflammation and stenosis of atherosclerotic plaque
***Hypercholesterolemia***						
Mandolini et al. 2015 [22]	Chieti (Italy)	N = 15 control N = 16 hypercholesterolemia	hsa-miR-33b; hsa-miR-758	upregulated in hypercholesterolemic group	tissue	hsa-miR-33b and hsa-miR-758 target ABCA1
Tanashyan et al. 2023 [23]	Moscow (Russia)	N = 26 control N = 35 hypercholesterolemia	hsa-miR-33a-5p and -3p	upregulated in hypercholesterolemic group	blood	cholesterol efflux by targeting the ABCA1
***Essential hypertension (EH)***						
Zhang et al. 2022 [24]	Ningbo (China)	N = 100 control N = 100 EH and CA	circ-0105130; circ-0109569; circ-0072659; circ-0079586; circ-0064684	upregulated in EH with carotid plaque	blood	possible target of hsa-miR-124 and hsa-miR-135a (in silico prediction)
Qian et al. 2023 [25]	Ningbo (China)	N = 64 control N = 64 EH N = 64 EH and CA	circ-0127342	downregulated in EH with carotid plaque	blood	circ-0127342 acts as sponge for hsa-miR-136-5p, hsa-miR-153-5p and hsa-miR-197-3p (bioinformatic analysis)
			circ-0124782; circ-0131618; circ-0127342	downregulated in EH with carotid plaque compared to healthy control		

**Table 2:** ncRNA correlated with carotid Intima-Media Thickness (cIMT).

Study and Reference	City and Country	Number of Participants	Comparisons	Source of ncRNA	ncRNA	Dysregulation
Minin et al. 2021 [26]	São Paulo (Brazil)	N = 72 IMT N = 105 CA	carotid plaque vs. IMT of carotid plaque in hypertensive patients	serum	hsa-miR-145-5p; hsa-miR-let7c	upregulated in carotid plaque group
Tao et al. 2022 [27]	Shanghai (China)	N = 90 control N = 95 IMT	asymptomatic CA patients vs. healthy patients	serum	SOX2-OT	upregulated in IMT group
Yan et al. 2024 [28]	Beijing (China)	N = 131 CA N = 119 IMT N = 123 controls	carotid plaque vs. IMT in hypertensive patients	blood (PBMCs)	circ-0043621	upregulated in CA compared to IMT; upregulated in IMT compared to control

**Table 3:** Differentially expressed ncRNA in carotid atherosclerosis versus healthy controls.

Study and Reference	City and Country	Number of Participants	Source of ncRNA	ncRNA	Dysregulation	Regulated Process
Huang et al. 2020 [29]	Deyang (China)	N = 90 control N = 180 CA	peripheral blood (PBMC)	hsa-miR-146a	upregulated in CA	Inflammation
Aroca-Esteban et al. 2024 [21]	Madrid (Spain)	N = 7 control N = 19 CA	EV (from plasma)	let-7d-5p	upregulated in CA	Inflammation
Jeong et al. 2021 [30]	Seoul (Korea)	N = 6 control N = 50 CA	plasma	hsa-miR-33a-5p; hsa-miR-33b-5p; hsa-miR-148a-3p	upregulated in CA	Cholesterol efflux
Tsai et al. 2013 [31]	Kaohsiung and Taichung (Taiwan)	N = 157 control N = 66 CA	serum	hsa-miR-21	upregulated in CA	VSMC proliferation
Liu et al. 2024 [32]	Shanghai (China)	N = 5 control N = 23 CA	tissue	hsa-miR-127-3p	upregulated in CA	Inflammation
Magenta et al. 2018 [33]	Rome (Italy)	N = 19 control N = 24 CA	plasma	hsa-miR-200c; hsa-miR-33a; hsa-miR-33b	upregulated in CA	Endothelial dysfunction
		N = 10 arterioles N = 22 plaque	tissue	hsa-miR-200c; hsa-miR-33a; hsa-miR-33b	upregulated in plaque	
Zhu et al. 2022 [34]	Hangzhou (China)	N = 25 control N = 52 CA	serum	hsa-miR-135a, hsa-miR-137, hsa-miR-149, hsa-miR-219a	upregulated in CA	-
				hsa-miR-126, hsa-miR-223, hsa-miR-101, hsa-miR-577, hsa-miR-384, hsa-miR-148	downregulated in CA	
Raskurazhev et al. 2020 [35]	Moscow (Russia)	N = 11 control N = 25 CA	serum	hsa-miR-33a	upregulated in CA	Cholesterol efflux
				hsa-miR-126-3p; hsa-miR-126-5p; miR21-3p; hsa-miR-21-5p	downregulated in CA	Inflammation and shear stress
Markus et al. 2016 [36]	Marburg (Germany)	N = 15 control N = 24 CA	tissue	hsa-miR-19b; hsa-miR-21; hsa-miR-22; hsa-miR-143	upregulated in CA (asymptomatic patients)	Macrophage infiltration and foam cell formation
				hsa-miR-1; hsa-miR-29b; let-7f	downregulated in CA	
Zhang et al. 2016 [37]	Tianjin (China)	N = 155 control N = 177 CA	serum	hsa-miR-320b	downregulated in CA	-
Luque et al. 2019 [38]	Barcelona (Spain)	N = 36 control N = 22 CA	serum	hsa-miR-638	downregulated in CA (symptomatic patients)	VSMC migration and proliferation
Han et al. 2018 [39]	Harbin (China)	N = 50 control N = 37 CA	plasma and tissue	hsa-miR-145	downregulated in CA	VSMC proliferation
Zhang et al. 2017 [40]	Jinan (China)	N = 46 control N = 46 plaque	tissue	hsa-miR-148b	downregulated in plaque	Endothelial dysfunction
Eken et al. 2017 [41]	Stockholm (Sweden)	N = 7 control N = 7 plaque	tissue	hsa-miR-210	downregulated in plaque	VSMC proliferation
Tian et al. 2023 [42]	Harbin (China)	N = 9 control N = 18 CA	blood (PBMC)	lncRNA AC078850.1	upregulated in CA	Inflammation
Fasolo et al. 2021 [43]	Stockholm (Sweden)	N = 13 control N = 77 plaque	tissue	MIAT	upregulated in plaque	VSMC proliferation, macrophages trans differentiation, inflammation
Huang et al. 2020 [44]	Wenzhou (China)	N = 60 control N = 60 CA	serum	CCAT2	upregulated in CA	-
				hsa-miR-216b	downregulated in CA	
Weng et al. 2021 [45]	Hainan (China)	N = 33control N = 35 CA	serum	LINC01123	upregulated in CA	VSMC migration and proliferation
				hsa-miR-1277-5p	downregulated in CA	
		N = 8 normal artery N = 8 plaque	tissue	LINC01123	upregulated in plaque	
Huang et al. 2016 [46]	Canton (China)	N = 5 normal renal artery N = 5 plaque	tissue	HOXC-AS1	downregulated in plaque	Foam cells formation
Lou et al. 2019 [47]	Ankang (China)	N = 3 control N = 5 CA	tissue	SENCR	downregulated in CA	Endothelial to mesenchymal transition
				hsa-miR-126a	upregulated in CA	
Wu et al. 2022 [48]	Shanghai (China)	N = 50 control N = 54 CA	serum	lncRNA FGF7-5; lncRNA GLRX3	downregulated in CA	Endothelial dysfunction
				hsa-miR-2681-5p	upregulated in CA	
Yan et al. 2024 [28]	Beijing (China)	N = 50 control N = 50 CA	blood (PBMCs)	circ-0043621; circ-0051995; circ-123388	upregulated in CA	Inflammation
				hsa-miR-223-3p	downregulated in CA	

**Table 4:** ncRNA and carotid plaque features.

Study and Reference	City and Country	Number of Participants	Source of ncRNA	ncRNA	Dysregulation	Plaque’s Features
***Regional differences***						
Raju et al. 2024 [49]	Toronto (Canada)	N = 20 (paired: plaque and marginal zones)	tissue (EV)	hsa-miR-146a, hsa-miR-155, let-7a, hsa-miR-200b, hsa-miR-223, hsa-miR-181b	upregulated in plaque	fibroatheroma and calcification in all plaque samples. Plaque zones contained more macrophages (EV source), while VSMC predominate in marginal zones. EVs per milligram of tissue compared to their matched marginal zones
Yan et al. 2024 [28]	Beijing (China)	N = 16 (paired: plaque vs. proximal adjacent region)	tissue	circ-0043621	upregulated in plaque	-
				hsa-miR-223-3p	downregulated in plaque	
Jeong et al. 2021 [30]	Seoul (Korea)	N = 50 (paired: internal vs. common carotid region)	tissue	hsa-miR-148a-3p	upregulated in internal carotid	The internal carotid artery exhibited accumulated plaque and shrunken arterial walls compared with the common carotid artery
***Stenosis severity***						
Huang et al. 2020 [29]	Deyang (China)	N = 64 mild N = 62 moderate N = 54 severe	peripheral blood (PBMC)	hsa-miR-146a	upregulated as the degrees of CAS stenosis increases	-
Raskurazhev et al. 2022 [50]	Moscow (Russia)	N = 31 moderate N = 30 advanced	blood (leukocytes)	hsa-miR-126-5p/3p; hsa-miR-21-5p/3p; hsa-miR-29-3p	downregulated in advanced CA	-
				hsa-miR-33a-5p/3p	upregulated in advanced CA	
Stenosis progression						
Dolz et al. 2017 [51]	Valencia (Spain)	N = 19 with stenosis progression N = 41 without stenosis progression	plasma (exosomes)	hsa-miR-199b-3p; hsa-miR-130a-3p; hsa-miR-24-3p	upregulated in ACAS progression group	-
***Plaque stability***						
Wang et al. 2020 [52]	Linyi (China)	N = 73 stable N = 87 vulnerable	serum	hsa-miR-124	upregulated in vulnerable plaque group	stable: fibrous and calcified plaque; vulnerable: lipid and mixed plaque
Huang et al. 2020 [29]	Deyang (China)	N = 96 stable N = 84 vulnerable	peripheral blood (PBMC)	hsa-miR-146a	upregulated in vulnerable plaque	-
Yang et al. 2018 [53]	Wuhan (China)	N = 13 stable N = 13 vulnerable	plasma	hsa-miR-23a-5p; hsa-miR-320a; hsa-miR-2110; hsa-miR-134-5p	upregulated in vulnerable plaque	-
				hsa-miR-4439	downregulated in vulnerable plaque	
Huang et al. 2020 [54]	Chongqing (China)	N = 50 stable N = 50 vulnerable	tissue	hsa-miR-532-3p	downregulated in vulnerable plaque group	-
Zhang et al. 2016 [37]	Tianjin (China)	N = 156 stable N = 21 vulnerable	serum	hsa-miR-320b	downregulated in vulnerable plaque group	-
Vasuri et al. 2020 [55]	Bologna (Italy)	N = 19 calcific core N = 18 protruding nodules	tissue	hsa-miR-30a-5p; hsa-miR-30d	upregulated in protruding nodules	calcific core = heavy calcium deposits superimposed over necrotic lipid plaque cores. protruding nodules = concentric nodular calcifications eroding the arterial walls, regardless of the amount of lipids
Katano et al. 2018 [56]	Nagoya (Japan)	N = 5 highly calcified N = 5 low calcified	tissue	hsa-miR-4530; hsa-miR-133b; hsa-miR-1-3p	upregulated in low calcified plaques	Macroscopic hemorrhages were relatively more frequent in the low-calcified plaques compared with the high-calcified plaques. No difference found between the high- and low-calcified plaques concerning the degrees of stenoses.
Magenta et al. 2018 [33]	Rome (Italy)	N = 10 stable N = 12 unstable	tissue	hsa-miR-200c	upregulated in unstable plaque	-
Liu et al. 2024 [32]	Shanghai (China)	N = 12 stable N = 11 unstable	tissue	hsa-miR-127-3p	upregulated in unstable plaque	-
Wei et al. 2019 [57]	Shanghai (China)	N = 10 stable N = 10 unstable	tissue	hsa-miR-330-5p	upregulated in unstable plaque	-
Eken et al. 2017 [41]	Stockholm (Sweden)	N = 10 stable N = 7 unstable	tissue	hsa-miR-210; hsa-miR-21	downregulated in ruptured plaque	cap thickness below (unstable) or above (stable) 200 µm.
Zhu et al. 2022 [34]	Hangzhou (China)	N = 23 stable N = 29 unstable	serum	hsa-miR-126; hsa-miR-223	downregulated in unstable plaque	-
Badacz et al. 2018 [58]	Krakow (Poland)	N = 24 hypoechogenic N = 47 moderately echogenic	serum	hsa-miR-124-3p; hsa-miR-134-5p; hsa-miR-34a-5p; hsa-miR-375	downregulated in hypoechogenic	hypoechogenic (or echolucent): soft, lipid rich (unstable) moderately echogenic: heterogeneous hyperechogenic: fibrotic and calcified (stable)
				hsa-miR-133b	upregulated in hypoechogenic	
		N = 47 moderately echogenic N = 21 hyperechogenic		hsa-miR-134-5p; hsa-miR-34a-5p; hsa-miR-375	upregulated in hyperechogenic	
				hsa-miR-16-5p	downregulated in hyperechogenic	
		N = 24 hypoechogenic N = 21 hyperechogenic		hsa-miR-16-5p	upregulated in hyperechogenic	
		N = 64 non-ulcerated N = 28 ulcerated		hsa-miR-1-3p; hsa-miR-16-5p	upregulated in ulcerated	
Fasolo et al. 2021 [43]	Stockholm (Sweden)	N = 10 stable N = 10 unstable	tissue	MIAT	upregulated in unstable plaque	-
Huang et al. 2020 [44]	Wenzhou (China)	N = 60 stable N = 75 unstable	serum	CCAT2	upregulated in unstable plaque	-
				hsa-miR-216b	downregulated in unstable plaque	
Bao et al. 2021 [59]	Jilin (China)	N = 5 stable N = 5 unstable	tissue	ENST00000430222; ENST00000602895; circ-013041; circ-025902	upregulated in unstable plaque	-
				ENST00000631338; MSTRG18183; circ-054182; circ-037511	downregulated in unstable plaque	
Wen et al. 2021 [60]	Shenzhen (China)	N = 22 stable N = 20 unstable	serum (exosomes)	circRNA-0006896	upregulated in unstable plaque	-
Lin et al. 2023 [61]	Shanghai (China)	N = 3 stable N = 3 unstable	tissue	circ-0001523; circ-0008950; circ-0000571	upregulated in unstable plaque	-

**Table 5:** Symptomatic vs. asymptomatic patients.

Study and Reference	Country	Number of Participants	Source of ncRNA	ncRNA	Dysregulation	Proposed Mechanism
Grosse et al. 2021 [62]	Hannover (Germany)	N = 23 asymptomatic N = 21 symptomatic	plasma	hsa-miR-92a	upregulated in symptomatic	-
Jeong et al. 2021 [30]	Seoul (Korea)	N = 37 asymptomatic N = 13 symptomatic	plasma	hsa-miR-33a-5p; hsa-miR-148a-3p	upregulated in symptomatic	all these miRNAs target 3′ UTR ABCA1 transcript
Tanashyan et al. 2024 [63]	Moscow (Russia)	N = 47 asymptomatic N = 34 symptomatic	serum	hsa-miR-200c-3p; hsa-miR-106b-5p; hsa-miR-494-5p	upregulated in symptomatic	-
				hsa-miR-183-3p; hsa-miR-126-5p; hsa-miR-216-3p	downregulated in symptomatic	
Eken et al. 2017 [41]	Stockholm (Sweden)	N = 5 asymptomatic N = 7 symptomatic	tissue	hsa-miR-29c	upregulated in symptomatic	-
			plasma	hsa-miR-210	downregulated in symptomatic	hsa-miR-210 targets APC mRNA and stimulates canonical Wnt signaling in VSMCs.
Badacz et al. 2018 [58]	Krakow (Poland)	N = 27 asymptomatic N = 65 symptomatic	serum	hsa-miR-124-3p; hsa-miR-134-5p	upregulated in symptomatic	brain-derived miRNAs
				hsa-miR-133a-3p	downregulated in symptomatic	
Markus et al. 2016 [36]	Giessen (Germany)	N = 14 asymptomatic N = 10 symptomatic	tissue	hsa-miR-21; hsa-miR-143	downregulated in symptomatic	-
Caparosa et al. 2019 [64]	Pittsburgh (USA)	N = 9 asymptomatic N = 9 symptomatic	tissue	hsa-miR-214, hsa-miR-484, hsa-miR-942, hsa-miR-1287	downregulated in symptomatic patients	mRNA targets: APOD (hsa-miR-214); DACH1 (hsa-miR-484); GPR56 (hsa-miR-942)

**Table 6:** Association of ncRNAs with ischemic stroke.

Study and Reference	Country	Number of Participants	Source of ncRNA	ncRNA	Dysregulation	Proposed Mechanism
Wang et al. 2024 [20]	Luoyang (China)	N = 18 without cerebral stroke N = 22 with cerebral stroke	tissue	hsa-miR-29c	upregulated in cerebral stroke group	VSMC proliferation
Tsai et al. 2013 [31]	Kaohsiung and Taichung (Taiwan)	N = 157 control N = 167 stroke	serum	hsa-miR-21	upregulated in stroke group	hsa-miR-21 is involved in apoptosis inhibition and in VSMC proliferation targeting PDCD4, PTEN and PI3K/Akt genes
				hsa-miR-221	downregulated in stroke group	
Bazan et al. 2015 [65]	New Orleans (USA)	N = 31 asymptomatic N = 20 symptomatic N = 25 cerebrovascular event	tissue	hsa-miR-221; hsa-miR-222	downregulated in cerebrovascular event (urgent) group	both miRNAs target p27^Kip1^, promoting VSMC proliferation
Bazan et al. 2017 [66]	New Orleans (USA)	N = 24 asymptomatic N = 17 cerebrovascular event	serum	hsa-miR-221	downregulated in cerebrovascular event (urgent) group	hsa-miR-221 is negatively regulated by circ-284.
Luque et al. 2019 [38]	Barcelona (Spain)	N = 36 control N = 11 stroke	serum	hsa-miR-638	downregulated stroke patients	-
Wang et al. 2023 [67]	Jinan (China)	N = 67 control (no plaque) N = 73 plaque with low risk of cerebrovascular event N = 85 plaque with medium-high risk of cerebrovascular event	serum (exosomes)	circSCMH1	downregulated in MH-risk compared to control and L-risk	presence of interaction sites within circSCMH1 and hsa-miR-874 sequence (bioinformatic analysis)
				hsa-miR-874	upregulated in MH-risk compared to control and L-risk	
Bazan et al. 2017 [66]	New Orleans (USA)	N = 48 asymptomatic N = 41 cerebrovascular event	serum	circR-284	upregulated in urgent group	hsa-miR-221 is negatively regulated by circ-284.

## Data Availability

The original contributions presented in this study are included in the article/Supplementary Materials. Further inquiries can be directed to the corresponding author.

## Supplementary Materials

The supporting information can be downloaded at: https://www.mdpi.com/article/10.3390/ijms27021002/s1.

## Abbreviations

ABCA1	ATP Binding Cassette Subfamily A Member 1
APC	Adenomatous Polyposis Coli
CEA	carotid endarterectomy
cIMT	carotid Intima- Media Thickness
circRNAs	circular RNAs
COX2	cycloxigenase-2
GSEA	Gene Set Enrichment Analysis
HDL	high-density lipoproteins
HIF-1	Hypoxia Inducible Factor 1
hs-CRP	high-sensitivity C-reactive protein
ICAM	intracellular adhesion molecules
IL	interleukin
KLF5	Krueppel-like factor 5
LDL	low-density lipoproteins
lncRNAs	long non-coding RNAs
Lp PLA 2	phospholipase a2
LRNC	lipid-rich necrotic core
MCP-1	monocyte chemoattractant factor-1
miRNAs	microRNAs
MSigDB	Molecular Signatures Database
ncRNAs	non-coding RNAs
NLRP3	Nod-like receptor protein 3
NO	nitric oxide
OSF	Open Science Framework
PBMC	peripheral blood mononuclear cells
PICOS	Population, Intervention, Comparison, Outcomes, and Study Design
PRISMA	Preferred Reporting Items for Systematic Reviews and Meta-Analyses
RBPs	RNA-binding proteins
RISC	RNA-induced silencing complex
TNF-α	tumor necrosis factor-alpha
VCAM	vascular cell adhesion molecules
VSMC	vascular smooth muscle cells
