# Supplementary material for: Non-Coding RNA Profile in the Progression of Carotid Atherosclerosis: A Systematic Review
Source: Int J Mol Sci. 2026 Jan 19;27(2):1002. doi: 10.3390/ijms27021002 (PMC12842102; doi:10.3390/ijms27021002)
Supplement: Supplementary file 1 [file ijms-27-01002-s001.zip › ijms-4094429-supplementary.pdf]

PRISMA 2020 Checklist

Supplementary Table S1 - PRISMA 2020 Checklist

Section and Topic	Item #	Checklist item	Location where item is reported
**TITLE**			
Title	1	Identify the report as a systematic review.	1
**ABSTRACT**			
Abstract	2	See the PRISMA 2020 for Abstracts checklist.	2
**INTRODUCTION**			
Rationale	3	Describe the rationale for the review in the context of existing knowledge.	3-4
Objectives	4	Provide an explicit statement of the objective(s) or question(s) the review addresses.	4
**METHODS**			
Eligibility criteria	5	Specify the inclusion and exclusion criteria for the review and how studies were grouped for the syntheses.	4-5
Information sources	6	Specify all databases, registers, websites, organisations, reference lists and other sources searched or consulted to identify studies. Specify the date when each source was last searched or consulted.	4-5
Search strategy	7	Present the full search strategies for all databases, registers and websites, including any filters and limits used.	4
Selection process	8	Specify the methods used to decide whether a study met the inclusion criteria of the review, including how many reviewers screened each record and each report retrieved, whether they worked independently, and if applicable, details of automation tools used in the process.	4-5, 7
Data collection process	9	Specify the methods used to collect data from reports, including how many reviewers collected data from each report, whether they worked independently, any processes for obtaining or confirming data from study investigators, and if applicable, details of automation tools used in the process.	5
Data items	10a	List and define all outcomes for which data were sought. Specify whether all results that were compatible with each outcome domain in each study were sought (e.g. for all measures, time points, analyses), and if not, the methods used to decide which results to collect.	5
	10b	List and define all other variables for which data were sought (e.g. participant and intervention characteristics, funding sources). Describe any assumptions made about any missing or unclear information.	5
Study risk of bias assessment	11	Specify the methods used to assess risk of bias in the included studies, including details of the tool(s) used, how many reviewers assessed each study and whether they worked independently, and if applicable, details of automation tools used in the process.	5
Effect measures	12	Specify for each outcome the effect measure(s) (e.g. risk ratio, mean difference) used in the synthesis or presentation of results.	-
Synthesis methods	13a	Describe the processes used to decide which studies were eligible for each synthesis (e.g. tabulating the study intervention characteristics and comparing against the planned groups for each synthesis (item #5)).	5
	13b	Describe any methods required to prepare the data for presentation or synthesis, such as handling of missing summary statistics, or data conversions.	5
	13c	Describe any methods used to tabulate or visually display results of individual studies and syntheses.	5
	13d	Describe any methods used to synthesize results and provide a rationale for the choice(s). If meta-analysis was performed, describe the	5

		model(s), method(s) to identify the presence and extent of statistical heterogeneity, and software package(s) used.	
	13e	Describe any methods used to explore possible causes of heterogeneity among study results (e.g. subgroup analysis, meta-regression).	-
	13f	Describe any sensitivity analyses conducted to assess robustness of the synthesized results.	-
Reporting bias assessment	14	Describe any methods used to assess risk of bias due to missing results in a synthesis (arising from reporting biases).	5
Certainty assessment	15	Describe any methods used to assess certainty (or confidence) in the body of evidence for an outcome.	5
**RESULTS**			
Study selection	16a	Describe the results of the search and selection process, from the number of records identified in the search to the number of studies included in the review, ideally using a flow diagram.	6-7
	16b	Cite studies that might appear to meet the inclusion criteria, but which were excluded, and explain why they were excluded.	7
Study characteristics	17	Cite each included study and present its characteristics.	7
Risk of bias in studies	18	Present assessments of risk of bias for each included study.	S(3-15)
Results of individual studies	19	For all outcomes, present, for each study: (a) summary statistics for each group (where appropriate) and (b) an effect estimate and its precision (e.g. confidence/credible interval), ideally using structured tables or plots.	-
Results of syntheses	20a	For each synthesis, briefly summarise the characteristics and risk of bias among contributing studies.	7-12
	20b	Present results of all statistical syntheses conducted. If meta-analysis was done, present for each the summary estimate and its precision (e.g. confidence/credible interval) and measures of statistical heterogeneity. If comparing groups, describe the direction of the effect.	-
	20c	Present results of all investigations of possible causes of heterogeneity among study results.	-
	20d	Present results of all sensitivity analyses conducted to assess the robustness of the synthesized results.	-
Reporting biases	21	Present assessments of risk of bias due to missing results (arising from reporting biases) for each synthesis assessed.	-
Certainty of evidence	22	Present assessments of certainty (or confidence) in the body of evidence for each outcome assessed.	-
**DISCUSSION**			
Discussion	23a	Provide a general interpretation of the results in the context of other evidence.	15-16
	23b	Discuss any limitations of the evidence included in the review.	16
	23c	Discuss any limitations of the review processes used.	-
	23d	Discuss implications of the results for practice, policy, and future research.	16
**OTHER INFORMATION**			
Registration and protocol	24a	Provide registration information for the review, including register name and registration number, or state that the review was not registered.	4
	24b	Indicate where the review protocol can be accessed, or state that a protocol was not prepared.	4-5
	24c	Describe and explain any amendments to information provided at registration or in the protocol.	-
Support	25	Describe sources of financial or non-financial support for the review, and the role of the funders or sponsors in the review.	16

Competing interests	26	Declare any competing interests of review authors.	16
Availability of data, code and other materials	27	Report which of the following are publicly available and where they can be found: template data collection forms; data extracted from included studies; data used for all analyses; analytic code; any other materials used in the review.	16

From: Page MJ, McKenzie JE, Bossuyt PM, Boutron I, Hoffmann TC, Mulrow CD, et al. The PRISMA 2020 statement: an updated guideline for reporting systematic reviews. BMJ 2021;372:n71. doi: 10.1136/bmj.n71
For more information, visit: http://www.prisma-statement.org/

PRISMA 2020 for Abstracts Checklist

Supplementary Table S2 - PRISMA 2020 for Abstracts Checklist

Section and Topic	Item #	Checklist item	Reported (Yes/No)
**TITLE**			
Title	1	Identify the report as a systematic review.	YES
**BACKGROUND**			
Objectives	2	Provide an explicit statement of the main objective(s) or question(s) the systematic review addresses.	YES
**METHODS**			
Eligibility criteria	3	Specify the inclusion and exclusion criteria for the systematic review.	YES
Information sources	4	Specify the information sources (e.g. databases, registers) used to identify studies and the date when each was last searched.	YES
Risk of bias	5	Specify the methods used to assess risk of bias in the included studies.	YES
Synthesis of results	6	Specify the methods used to present and synthesise results.	YES
**RESULTS**			
Included studies	7	Give the total number of included studies and participants and summarise relevant characteristics of studies.	YES
Synthesis of results	8	Present results for main outcomes, preferably indicating the number of included studies and participants for each. If systematic review was done, report the summary estimate and confidence/credible interval. If comparing groups, indicate the direction of the effect (i.e. which group is favoured).	YES
**DISCUSSION**			
Limitations of evidence	9	Provide a brief summary of the limitations of the evidence included in the systematic review (e.g. study risk of bias, inconsistency and imprecision).	YES
Interpretation	10	Provide a general interpretation of the results and important implications.	YES
**OTHER**			
Funding	11	Specify the primary source of funding for the systematic review.	YES
Registration	12	Provide the register name and registration number.	YES

From: Page MJ, McKenzie JE, Bossuyt PM, Boutron I, Hoffmann TC, Mulrow CD, et al. The PRISMA 2020 statement: an updated guideline for reporting systematic reviews. BMJ 2021;372:n71. doi: 10.1136/bmj.n71

For more information, visit: http://www.prisma-statement.org/

Supplementary Table S3– Keywords

KEY WORDS
Carotid atherosclerosis, Carotid plaque
noncoding RNA, ncRNA, microRNA, miRNA, long noncoding RNA, lncRNA, circular RNA, circRNA, PIWI-interacting RNA, piRNA, YRNA, tsRNA, extracellular RNA, exRNA

Supplementary Table S4 – Search Strategy

Database	Steps	Query	Research in	Items found
**PubMed**	#1	("Carotid atherosclerosis"[Title/Abstract]) OR ("Carotid plaque*"[Title/Abstract])	Title/Abstract	9,896
	#2	((((((((((((("noncoding RNA"[Title/Abstract]) OR (ncRNA[Title/Abstract])) OR (ncRNA[Title/Abstract])) OR (microRNA[Title/Abstract])) OR (miRNA[Title/Abstract])) OR (long noncoding RNA[Title/Abstract])) OR (lncRNA[Title/Abstract])) OR ("circular RNA"[Title/Abstract])) OR (circRNA[Title/Abstract])) OR ("PIWI-interacting RNA"[Title/Abstract])) OR (piRNA[Title/Abstract])) OR (YRNA[Title/Abstract])) OR (tsRNA[Title/Abstract])) OR ("extracellular RNA"[Title/Abstract])) OR (exRNA[Title/Abstract])	Title/Abstract	180,846
	#3	Combine #1 AND #2		81
	#4	Limit to (English) and (Italian)		80
**Scopus**	#1	TITLE-ABS-KEY ("Carotid atherosclerosis" OR "Carotid plaque*")	Title/Abstract/Keywords	15,460
	#2	TITLE-ABS-KEY ("noncoding RNA" OR "ncRNA" OR "microRNA" OR "miRNA" OR "long noncoding RNA" OR "lncRNA" OR "circular RNA" OR "circRNA" OR "PIWI-interacting RNA" OR "piRNA" OR "YRNA" OR "tsRNA" OR "extracellular RNA" OR "exRNA")	Title/Abstract/Keywords	277,514
	#3	Combine #1 AND #2		195
	#4	Limit to (English) and (Italian)		187

Supplementary Table S5 - Population, intervention, comparison, outcomes and study design (PICOS) worksheet.

Parameters	Inclusion Criteria	Exclusion Criteria
**Participants**	Studies in humans<br>Studies including patients with carotid atherosclerosis	In vitro, in vivo and in silico studies<br>Participants with other malignancies or pathologies
**Interventions**	Assessment of extracellular, circulating ncRNA expression, and<br>intracellular ncRNAs.	Others
**Comparisons**	Control Group (between or within design)	Other types of atherosclerosis (coronary, aortic, femoral)
**Outcomes**	1) To provide unbiased and exhaustive overview of the current knowledge on the ncRNAs profile and their mechanism of action in patients with carotid atherosclerosis<br>2) To summarize the major ncRNA associated with cognitive function in carotid atherosclerosis.	Others
**Study Design**	Original studies in English and Italian	Review, Scoping Review, Narrative Review, Systematic Review, Meta-Analysis, Editorial, Book, Case Report, Conference Review, and Conference Paper

Supplementary Table S6 - The Appraisal tool for Cross-Sectional Studies (AXIS tool, 2016)

**The Appraisal tool for Cross-Sectional Studies (AXIS tool, 2016) Part 1**								
Study	**Aroca-Esteban, et al. 2024**	**Badacz, et al. 2017**	**Bao, et al. 2021**	**Bazan, et al. 2015**	**Bazan, et al. 2017**	**Caparosa, et al. 2018**	**Dolz, et al. 2016**	**Eken, et al. 2016**
Introduction								
1. Were the aims/objectives of the study clear?	YES	YES	YES	YES	YES	YES	YES	YES
Methods								
2. Was the study design appropriate for the stated aim(s)?	YES	YES	YES	NO	YES	YES	YES	YES
3. Was the sample size justified?	NO	NO	NO	NO	NO	YES	NO	NO

4. Was the target/reference population clearly defined? (Is it clear who the research was about?)	YES	YES	YES	YES	YES	YES	YES	YES
5. Was the sample frame taken from an appropriate population base so that it closely represented the target/reference population under investigation?	YES	YES	YES	YES	YES	YES	YES	YES
6. Was the selection process likely to select subjects/participants that were representative of the target/reference population under investigation?	YES	YES	YES	YES	YES	YES	YES	YES
7. Were measures undertaken to address and categorise non-responders?	NOT APPROPRIATE	NOT APPROPRIATE	NOT APPROPRIATE	NOT APPROPRIATE	NOT APPROPRIATE	NOT APPROPRIATE	NOT APPROPRIATE	NOT APPROPRIATE
8. Were the risk factor and outcome variables measured appropriate to the aims of the study?	YES	YES	YES	YES	YES	YES	YES	YES
9. Were the risk factor and outcome variables measured correctly using instruments/ measurements that had been trialled, piloted or published previously?	YES	YES	YES	YES	YES	YES	YES	YES
10. Is it clear what was used to determined statistical significance and/or precision estimates? (eg, p values, CIs)	YES	YES	YES	YES	YES	YES	YES	YES

11. Were the methods (including statistical methods) sufficiently described to enable them to be repeated?	YES	YES	YES	NO	NO	YES	YES	YES
Results								
12. Were the basic data adequately described?	YES	YES	YES	YES	YES	YES	YES	NO
13. Does the response rate raise concerns about non-response bias?	NOT APPROPRIATE	NOT APPROPRIATE	NOT APPROPRIATE	NOT APPROPRIATE	NOT APPROPRIATE	NOT APPROPRIATE	NOT APPROPRIATE	NOT APPROPRIATE
14. If appropriate, was information about non-responders described?	NOT APPROPRIATE	NOT APPROPRIATE	NOT APPROPRIATE	NOT APPROPRIATE	NOT APPROPRIATE	NOT APPROPRIATE	NOT APPROPRIATE	NOT APPROPRIATE
15. Were the results internally consistent?	YES	YES	YES	YES	YES	YES	YES	YES
16. Were the results for the analyses described in the methods, presented?	YES	YES	YES	YES	YES	YES	YES	YES

**The Appraisal tool for Cross-Sectional Studies (AXIS tool, 2016) Part 2**								
Study	**Fasolo, et al. 2021**	**Grosse, et al. 2021**	**Han, et al. 2018**	**Huang et al. 2016**	**Huang et al. 2020 (ref.47)**	**Huang et al. 2020 (ref. 53)**	**Huang et al. 2020 (ref.72)**	**Jeong, et al. 2021**
Introduction								
1. Were the aims/objectives of the study clear?	YES	YES	YES	YES	YES	YES	YES	YES
Methods								
2. Was the study design appropriate for the stated aim(s)?	YES	YES	YES	YES	YES	YES	YES	YES
3. Was the sample size justified?	YES	YES	YES	NO	YES	NO	YES	NO
4. Was the target/reference population clearly	YES	YES	YES	YES	YES	YES	YES	YES

defined? (Is it clear who the research was about?)								
5. Was the sample frame taken from an appropriate population base so that it closely represented the target/reference population under investigation?	YES	YES	YES	YES	YES	YES	YES	YES
6. Was the selection process likely to select subjects/participants that were representative of the target/reference population under investigation?	YES	YES	YES	YES	YES	YES	YES	YES
7. Were measures undertaken to address and categorise non-responders?	NOT APPROPRIATE	NOT APPROPRIATE	NOT APPROPRIATE	NOT APPROPRIATE	NOT APPROPRIATE	NOT APPROPRIATE	NOT APPROPRIATE	NOT APPROPRIATE
8. Were the risk factor and outcome variables measured appropriate to the aims of the study?	YES	YES	YES	YES	YES	YES	YES	YES
9. Were the risk factor and outcome variables measured correctly using instruments/ measurements that had been trialled, piloted or published previously?	YES	YES	YES	YES	YES	YES	YES	YES
10. Is it clear what was used to determined statistical significance and/or precision estimates? (eg, p values, CIs)	YES	YES	YES	YES	YES	YES	YES	YES

11. Were the methods (including statistical methods) sufficiently described to enable them to be repeated?	YES	YES	YES	YES	YES	YES	YES	YES
Results								
12. Were the basic data adequately described?	YES	YES	YES	YES	NO	NO	YES	YES
13. Does the response rate raise concerns about non-response bias?	NOT APPROPRIATE	NOT APPROPRIATE	NOT APPROPRIATE	NOT APPROPRIATE	NOT APPROPRIATE	NOT APPROPRIATE	NOT APPROPRIATE	NOT APPROPRIATE
14. If appropriate, was information about non-responders described?	NOT APPROPRIATE	NOT APPROPRIATE	NOT APPROPRIATE	NOT APPROPRIATE	NOT APPROPRIATE	NOT APPROPRIATE	NOT APPROPRIATE	NOT APPROPRIATE
15. Were the results internally consistent?	YES	NO	YES	YES	YES	YES	YES	YES
16. Were the results for the analyses described in the methods, presented?	YES	YES	YES	YES	YES	YES	YES	YES

**The Appraisal tool for Cross-Sectional Studies (AXIS tool, 2016) Part 3**								
Study	**Katano, et al. 2018**	**Lin, et al. 2022**	**Liu, et al. 2024**	**Lou, et al. 2019**	**Luque, et al. 2019**	**Magenta, et al. 2018**	**Mandolini, et al. 2015**	**Markus, et al. 2016**
Introduction								
1. Were the aims/objectives of the study clear?	YES	YES	YES	YES	YES	YES	YES	YES
Methods								
2. Was the study design appropriate for the stated aim(s)?	YES	YES	YES	NO	YES	YES	YES	YES
3. Was the sample size justified?	NO	NO	NO	NO	YES	NO	YES	NO

4. Was the target/reference population clearly defined? (Is it clear who the research was about?)	YES	YES	YES	YES	YES	YES	YES	YES
5. Was the sample frame taken from an appropriate population base so that it closely represented the target/reference population under investigation?	YES	YES	YES	YES	YES	YES	YES	YES
6. Was the selection process likely to select subjects/participants that were representative of the target/reference population under investigation?	YES	YES	YES	NO	YES	YES	YES	YES
7. Were measures undertaken to address and categorise non-responders?	NOT APPROPRIATE	NOT APPROPRIATE	NOT APPROPRIATE	NOT APPROPRIATE	NOT APPROPRIATE	NOT APPROPRIATE	NOT APPROPRIATE	NOT APPROPRIATE
8. Were the risk factor and outcome variables measured appropriate to the aims of the study?	YES	YES	YES	YES	YES	YES	YES	YES
9. Were the risk factor and outcome variables measured correctly using instruments/ measurements that had been trialled, piloted or published previously?	YES	YES	YES	YES	YES	YES	YES	YES
10. Is it clear what was used to determined statistical significance and/or precision estimates? (eg, p values, CIs)	YES	YES	YES	YES	YES	YES	YES	YES

11. Were the methods (including statistical methods) sufficiently described to enable them to be repeated?	YES	YES	YES	YES	YES	YES	YES	YES
Results								
12. Were the basic data adequately described?	YES	YES	YES	YES	YES	YES	YES	YES
13. Does the response rate raise concerns about non-response bias?	NOT APPROPRIATE	NOT APPROPRIATE	NOT APPROPRIATE	NOT APPROPRIATE	NOT APPROPRIATE	NOT APPROPRIATE	NOT APPROPRIATE	NOT APPROPRIATE
14. If appropriate, was information about non-responders described?	NOT APPROPRIATE	NOT APPROPRIATE	NOT APPROPRIATE	NOT APPROPRIATE	NOT APPROPRIATE	NOT APPROPRIATE	NOT APPROPRIATE	NOT APPROPRIATE
15. Were the results internally consistent?	YES	YES	YES	YES	YES	YES	YES	YES
16. Were the results for the analyses described in the methods, presented?	YES	YES	YES	YES	YES	YES	YES	YES

**The Appraisal tool for Cross-Sectional Studies (AXIS tool, 2016) Part 4**								
Study	**Minin, et al. 2021**	**Qian, et al. 2023**	**Raju, et al. 2024**	**Raskurazhev, et al. 2020**	**Raskurazhev, et al. 2022**	**Tanashyan, et al. 2023**	**Tanashyan, et al. 2024**	**Tao, et al. 2022**
Introduction								
1. Were the aims/objectives of the study clear?	YES	YES	YES	YES	YES	YES	YES	YES
Methods								
2. Was the study design appropriate for the stated aim(s)?	YES	YES	YES	YES	YES	YES	YES	YES
3. Was the sample size justified?	YES	NO	NO	NO	YES	YES	YES	NO
4. Was the target/reference population clearly defined? (Is it clear	YES	YES	YES	YES	YES	YES	YES	YES

who the research was about?)								
5. Was the sample frame taken from an appropriate population base so that it closely represented the target/reference population under investigation?	YES	YES	YES	YES	YES	YES	YES	YES
6. Was the selection process likely to select subjects/participants that were representative of the target/reference population under investigation?	YES	YES	YES	YES	YES	YES	YES	YES
7. Were measures undertaken to address and categorise non-responders?	NOT APPROPRIATE	NOT APPROPRIATE	NOT APPROPRIATE	NOT APPROPRIATE	NOT APPROPRIATE	NOT APPROPRIATE	NOT APPROPRIATE	NOT APPROPRIATE
8. Were the risk factor and outcome variables measured appropriate to the aims of the study?	YES	YES	YES	YES	YES	YES	YES	YES
9. Were the risk factor and outcome variables measured correctly using instruments/ measurements that had been trialled, piloted or published previously?	YES	YES	YES	YES	YES	YES	YES	YES
10. Is it clear what was used to determined statistical significance and/or precision estimates? (eg, p values, CIs)	YES	YES	YES	NO	YES	YES	YES	YES
11. Were the methods (including statistical methods) sufficiently described to enable	YES	YES	YES	YES	YES	YES	YES	YES

them to be repeated?								
Results								
12. Were the basic data adequately described?	YES	YES	YES	NO	YES	YES	YES	YES
13. Does the response rate raise concerns about non-response bias?	NOT APPROPRIATE	NOT APPROPRIATE	NOT APPROPRIATE	NOT APPROPRIATE	NOT APPROPRIATE	NOT APPROPRIATE	NOT APPROPRIATE	NOT APPROPRIATE
14. If appropriate, was information about non-responders described?	NOT APPROPRIATE	NOT APPROPRIATE	NOT APPROPRIATE	NOT APPROPRIATE	NOT APPROPRIATE	NOT APPROPRIATE	NOT APPROPRIATE	NOT APPROPRIATE
15. Were the results internally consistent?	YES	YES	YES	YES	YES	YES	YES	YES
16. Were the results for the analyses described in the methods, presented?	YES	YES	YES	YES	YES	YES	YES	YES

**The Appraisal tool for Cross-Sectional Studies (AXIS tool, 2016) Part 5**								
Study	**Tian, et al. 2023**	**Tsai, et al. 2012**	**Vasuri, et al. 2019**	**Wang, et al. 2020**	**Wang, et al. 2023**	**Wang, et al. 2024**	**Wei, et al. 2019**	**Wen, et al. 2021**
Introduction								
1. Were the aims/objectives of the study clear?	YES	YES	YES	YES	YES	YES	YES	YES
Methods								
2. Was the study design appropriate for the stated aim(s)?	YES	YES	YES	YES	YES	YES	YES	YES
3. Was the sample size justified?	NO	YES	YES	YES	YES	NO	YES	YES
4. Was the target/reference population clearly defined? (Is it clear who the research was about?)	YES	YES	YES	YES	YES	YES	YES	YES
5. Was the sample frame taken from an appropriate	YES	YES	YES	YES	YES	YES	YES	YES

population base so that it closely represented the target/reference population under investigation?								
6. Was the selection process likely to select subjects/participants that were representative of the target/reference population under investigation?	YES	YES	YES	YES	YES	YES	YES	YES
7. Were measures undertaken to address and categorise non-responders?	NOT APPROPRIATE	NOT APPROPRIATE	NOT APPROPRIATE	NOT APPROPRIATE	NOT APPROPRIATE	NOT APPROPRIATE	NOT APPROPRIATE	NOT APPROPRIATE
8. Were the risk factor and outcome variables measured appropriate to the aims of the study?	YES	YES	YES	YES	YES	YES	YES	YES
9. Were the risk factor and outcome variables measured correctly using instruments/ measurements that had been trialled, piloted or published previously?	YES	YES	YES	YES	YES	YES	YES	YES
10. Is it clear what was used to determined statistical significance and/or precision estimates? (eg, p values, CIs)	YES	YES	YES	YES	YES	YES	YES	YES
11. Were the methods (including statistical methods) sufficiently described to enable them to be repeated?	YES	YES	YES	YES	YES	YES	YES	YES
Results								

12. Were the basic data adequately described?	YES	YES	YES	YES	YES	YES	YES	YES
13. Does the response rate raise concerns about non-response bias?	NOT APPROPRIATE	NOT APPROPRIATE	NOT APPROPRIATE	NOT APPROPRIATE	NOT APPROPRIATE	NOT APPROPRIATE	NOT APPROPRIATE	NOT APPROPRIATE
14. If appropriate, was information about non-responders described?	NOT APPROPRIATE	NOT APPROPRIATE	NOT APPROPRIATE	NOT APPROPRIATE	NOT APPROPRIATE	NOT APPROPRIATE	NOT APPROPRIATE	NOT APPROPRIATE
15. Were the results internally consistent?	YES	YES	YES	YES	YES	YES	YES	YES
16. Were the results for the analyses described in the methods, presented?	YES	YES	YES	YES	YES	YES	YES	YES

**The Appraisal tool for Cross-Sectional Studies (AXIS tool, 2016) Part 6**									
Study	**Weng, et al. 2020**	**Wu, et al. 2021**	**Yan, et al. 2024**	**Yang, et al. 2018**	**Yang, et al. 2021**	**Zhang, et al. 2016**	**Zhang, et al. 2017**	**Zhang, et al. 2022**	**Zhu, et al. 2021**
Introduction									
1. Were the aims/objectives of the study clear?	YES	YES	YES	YES	YES	YES	YES	YES	YES
Methods									
2. Was the study design appropriate for the stated aim(s)?	YES	YES	YES	YES	YES	YES	YES	YES	YES
3. Was the sample size justified?	YES	NO	YES	YES	NO	NO	NO	YES	YES
4. Was the target/reference population clearly defined? (Is it clear who the research was about?)	YES	YES	YES	YES	YES	YES	NO	YES	YES
5. Was the sample frame taken from an appropriate	YES	YES	YES	YES	YES	YES	YES	YES	YES

population base so that it closely represented the target/reference population under investigation?									
6. Was the selection process likely to select subjects/participants that were representative of the target/reference population under investigation?	YES	YES	YES	YES	NO	YES	YES	YES	YES
7. Were measures undertaken to address and categorise non-responders?	NOT APPROPRIATE	NOT APPROPRIATE	NOT APPROPRIATE	NOT APPROPRIATE	NOT APPROPRIATE	NOT APPROPRIATE	NOT APPROPRIATE	NOT APPROPRIATE	NOT APPROPRIATE
8. Were the risk factor and outcome variables measured appropriate to the aims of the study?	YES	YES	YES	YES	YES	YES	YES	YES	YES
9. Were the risk factor and outcome variables measured correctly using instruments/ measurements that had been trialled, piloted or published previously?	YES	YES	YES	YES	YES	YES	YES	YES	YES
10. Is it clear what was used to determined statistical significance and/or precision estimates? (eg, p values, CIs)	YES	YES	YES	YES	YES	YES	YES	YES	YES
11. Were the methods (including statistical methods) sufficiently	YES	YES	YES	YES	YES	YES	YES	YES	YES

described to enable them to be repeated?									
Results									
12. Were the basic data adequately described?	YES	YES	YES	YES	YES	YES	YES	YES	YES
13. Does the response rate raise concerns about non-response bias?	NOT APPROPRIATE	NOT APPROPRIATE	NOT APPROPRIATE	NOT APPROPRIATE	NOT APPROPRIATE	NOT APPROPRIATE	NOT APPROPRIATE	NOT APPROPRIATE	NOT APPROPRIATE
14. If appropriate, was information about non-responders described?	NOT APPROPRIATE	NOT APPROPRIATE	NOT APPROPRIATE	NOT APPROPRIATE	NOT APPROPRIATE	NOT APPROPRIATE	NOT APPROPRIATE	NOT APPROPRIATE	NOT APPROPRIATE
15. Were the results internally consistent?	YES	YES	YES	YES	YES	YES	YES	YES	YES
16. Were the results for the analyses described in the methods, presented?	YES	YES	YES	YES	YES	YES	YES	YES	YES

Supplementary Table S7 - Functional groups of atherosclerosis-related biological processes with corresponding GO identifier.

GO ID	Biological process
Inflammation and immune response	
GO:0006954	Regulation of inflammatory response
GO:0002526	Cellular response to interleukin-6
GO:0071347	Cellular response to interleukin-1
GO:0019221	Cytokine-mediated signaling pathway
GO:0002250	Regulation of chronic inflammatory response
GO:0002367	Regulation of interleukin-10 production
Oxidative stress	
GO:0000302	Response to reactive oxygen species
GO:1903200	Regulation of oxidative stress-induced cell death
GO:0006979	Response to oxidative stress
GO:0098869	Cellular oxidant detoxification

Endothelial and vascular response	
GO:0001936	Regulation of endothelial cell proliferation
GO:0007596	Regulation of leukocyte adhesion to vascular endothelial cell
GO:0050817	Coagulation
GO:1904706	Regulation of vascular associated smooth muscle cell migration
GO:1904707	Regulation of vascular associated smooth muscle cell proliferation
GO:0043114	Regulation of vascular permeability
GO:0034405	Response to fluid shear stress
Lipid metabolism	
GO:0006869	Lipid transport
GO:0055088	Lipoprotein transport
GO:0034375	High-density lipoprotein particle clearance
GO:0034383	Low-density lipoprotein particle clearance
GO:0034380	Regulation of cholesterol transport
GO:0042157	Lipoprotein metabolic process
GO:0090181	Regulation of cholesterol metabolic process
GO:0045588	Regulation of cholesterol biosynthetic process
GO:0090178	Regulation of cholesterol efflux
GO:0043691	Reverse cholesterol transport
Plaque formation and foam cells	
GO:0070483	Regulation of monocyte aggregation
GO:2000477	Regulation of monocyte chemotaxis
GO:0002525	Regulation of monocyte differentiation
GO:0002548	Regulation of monocyte extravasation
GO:0071404	Cellular response to lipoprotein particle stimulus
GO:0034378	Lipoprotein particle mediated signaling
GO:0034381	Plasma lipoprotein particle clearance
GO:0002438	Plasma lipoprotein particle remodeling
GO:0010886	Positive regulation of macrophage derived foam cell differentiation
